# Supplementary material for: HFIP-Promoted Bischler Indole Synthesis under Microwave Irradiation
Source: Molecules. 2018 Dec 14;23(12):3317. doi: 10.3390/molecules23123317 (PMC6321335; doi:10.3390/molecules23123317)

Supporting Information

HFIP-Promoted Bischler Indole Synthesis under Microwave Irradiation

Guangkai Yao [1,3], Zhi-Xiang Zhang [1,3], Cheng-Bei Zhang[1,3], Han-Hong Xu [1,3]* and Ri-Yuan Tang [1,2]*

[1] Key Laboratory of Natural Pesticide and Chemical Biology, Ministry of Education, South China Agricultural University, Guangzhou 510642, China;

[2] Department of Applied Chemistry, College of Materials and Energy, South China Agricultural University, Guangzhou 510642, China;

[3] State Key Laboratory for Conservation and Utilization of Subtropical Agro-Bioresources, South China Agricultural University, Guangzhou 510642, China;

* Correspondence: hhxu@scau.edu.cn (H.-H.X.); rytang@scau.edu.cn (R.-Y.T.)

Tables of Contents

Copies of ^{1}H NMR and ^{13}CNMR Spectra-- S1-S32

^{1}H and ^{13}C-NMR Spectra

1-Methyl-3-phenyl-1*H*-indole (**2**)

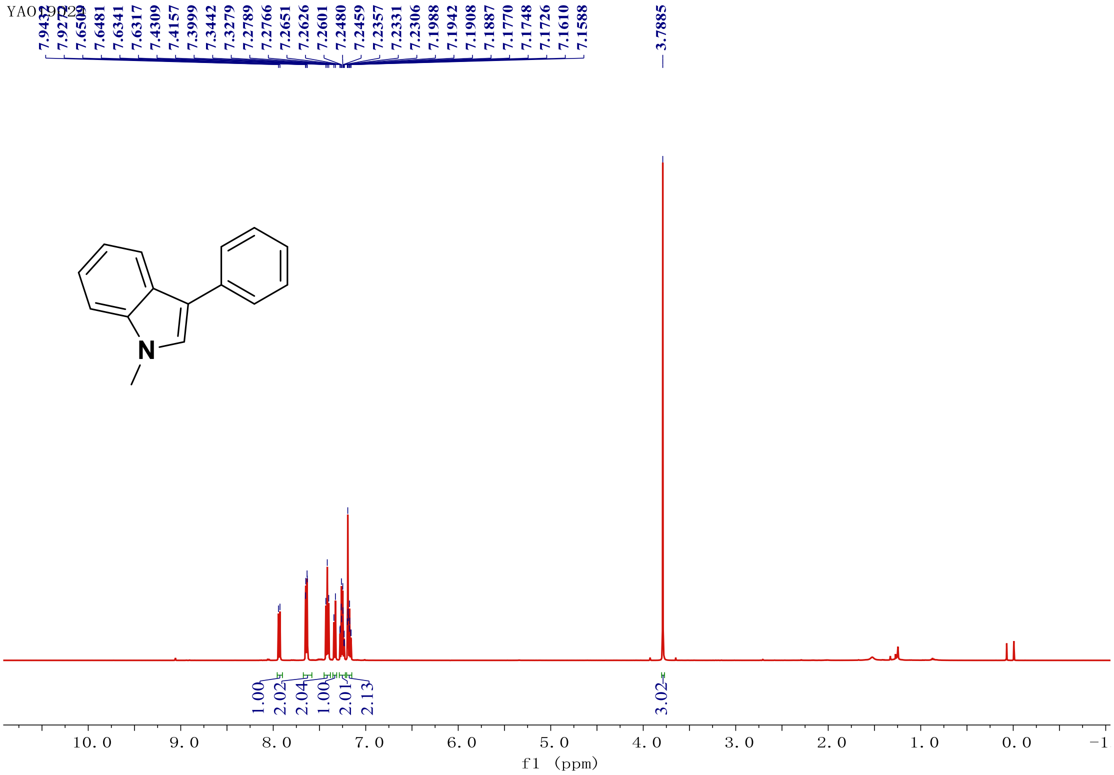

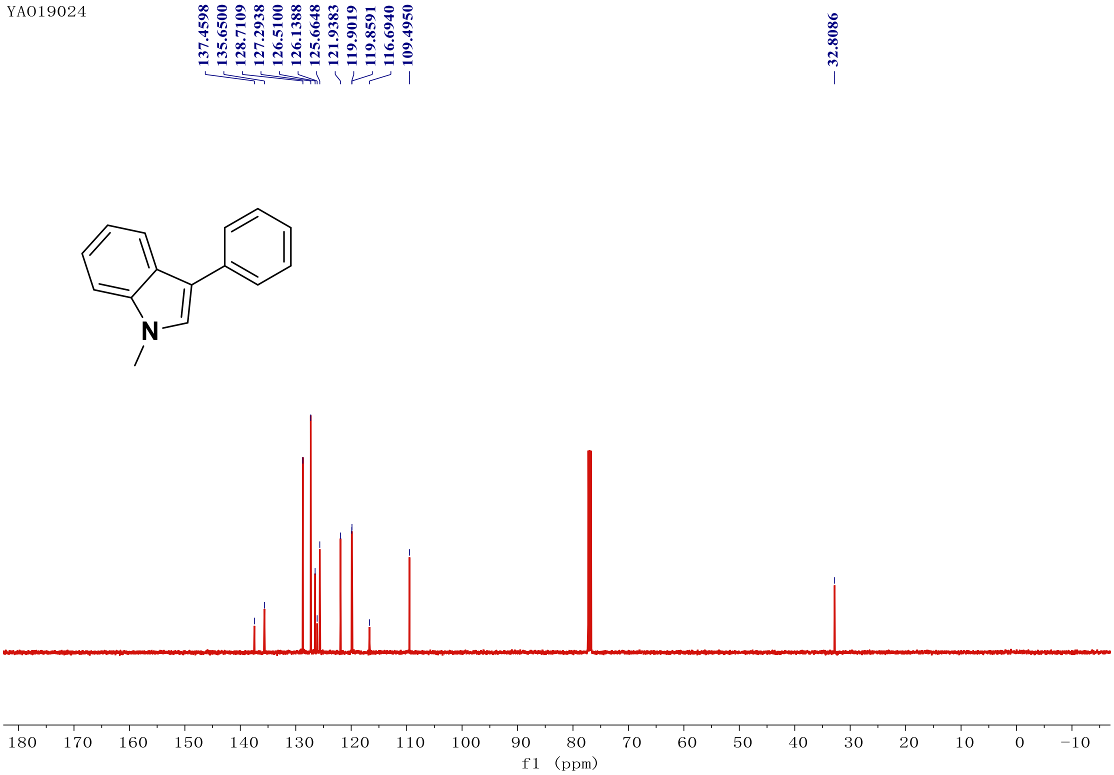

1-Methyl-3-(*p*-tolyl)-1*H*-indole (**3**)

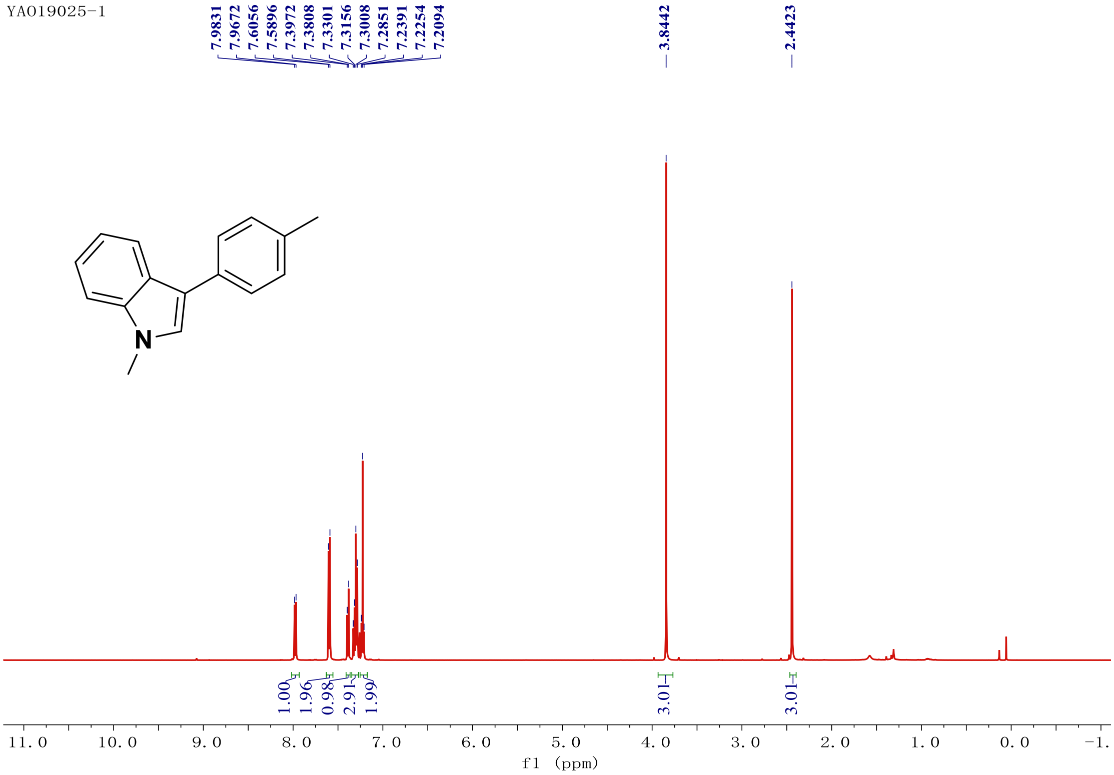

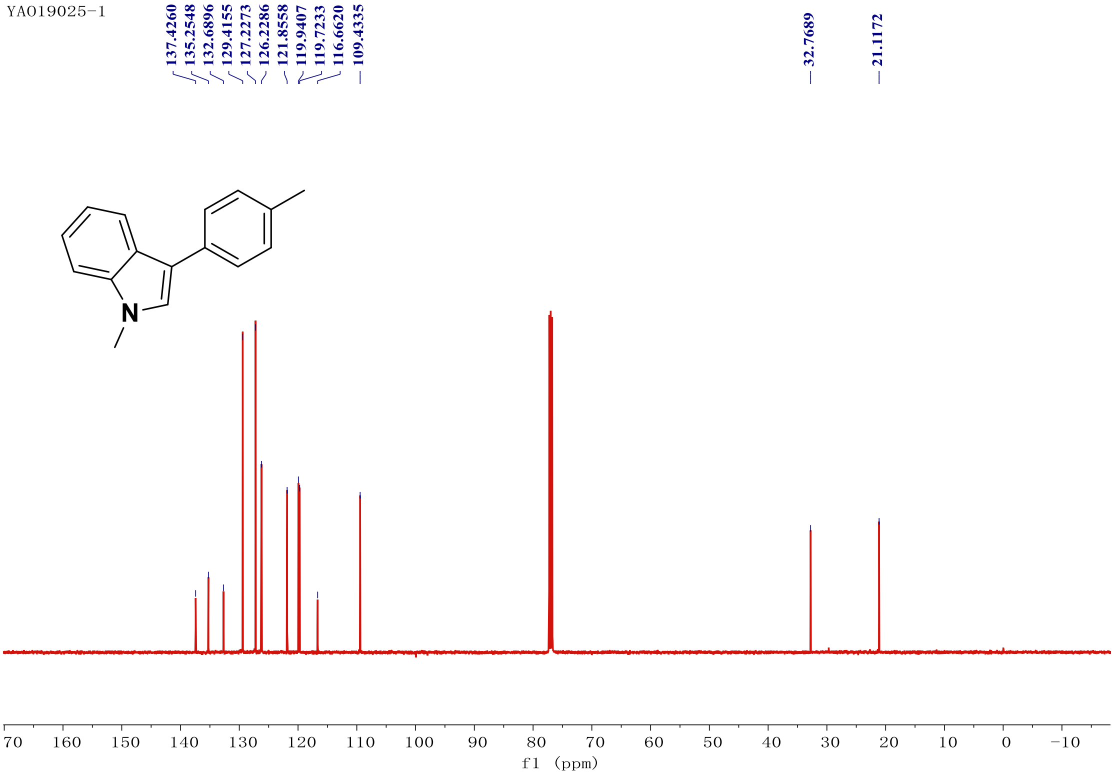

3-([1,1'-Biphenyl]-4-yl)-1-methyl-1*H*-indole (**4**)

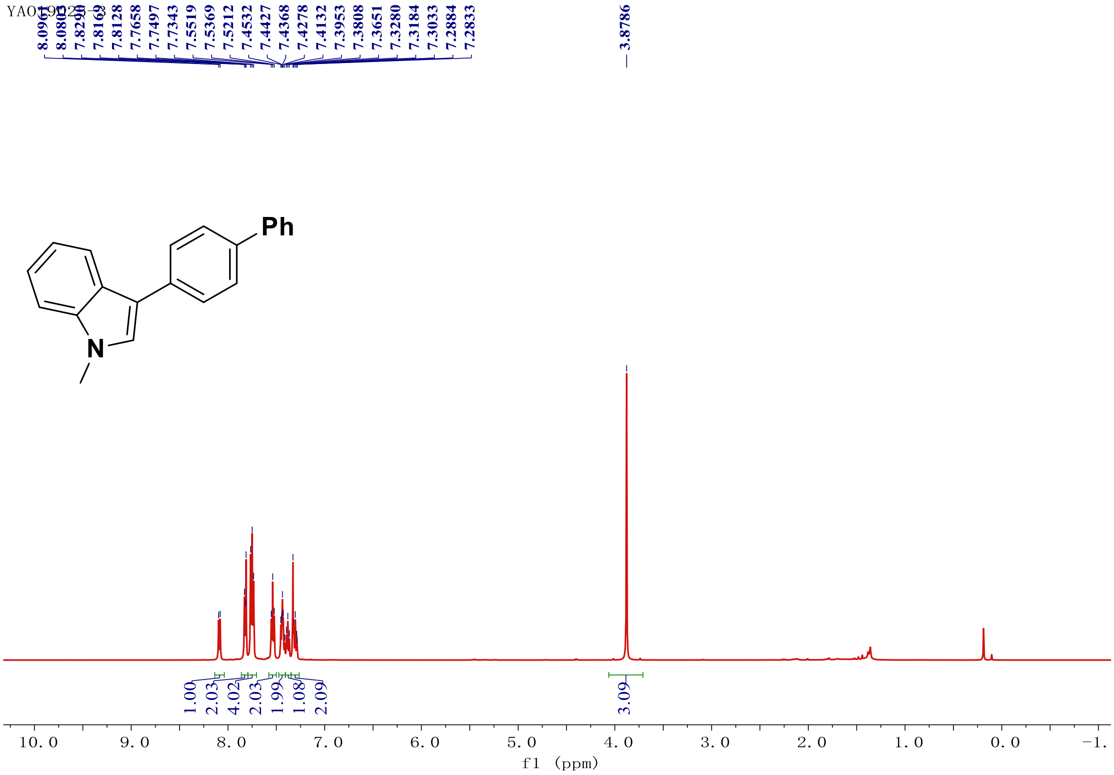

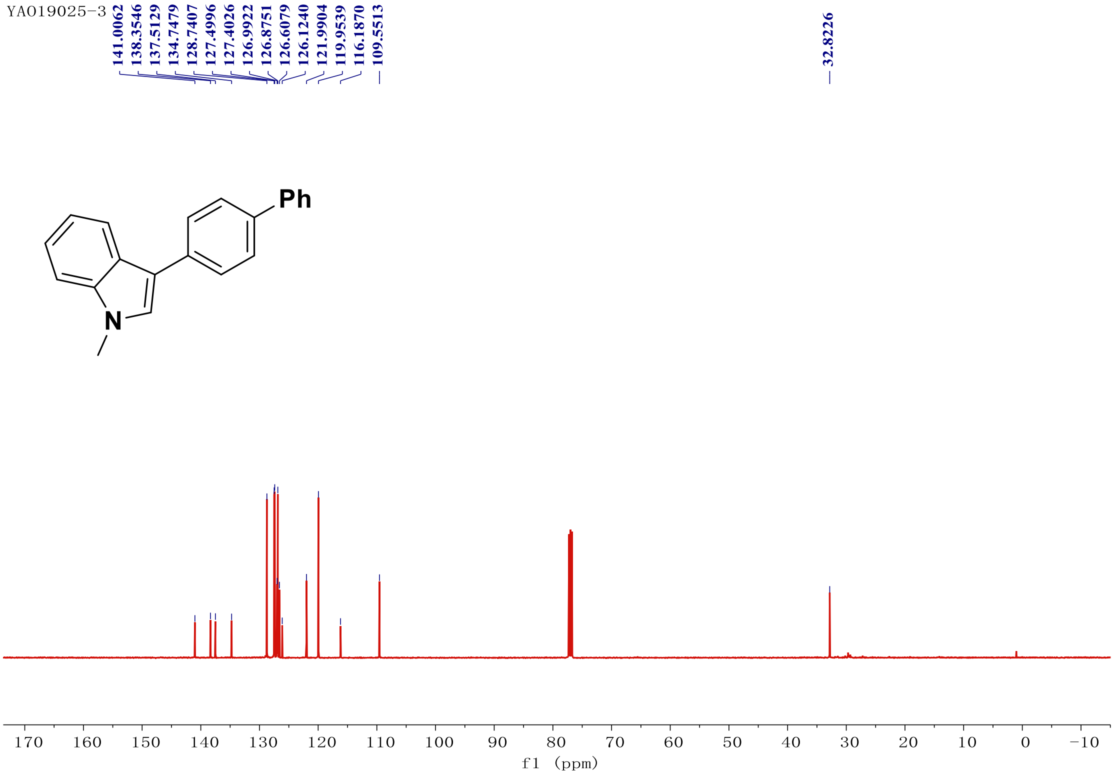

3-(4-Methoxyphenyl)-1-methyl-1*H*-indole (**5**)

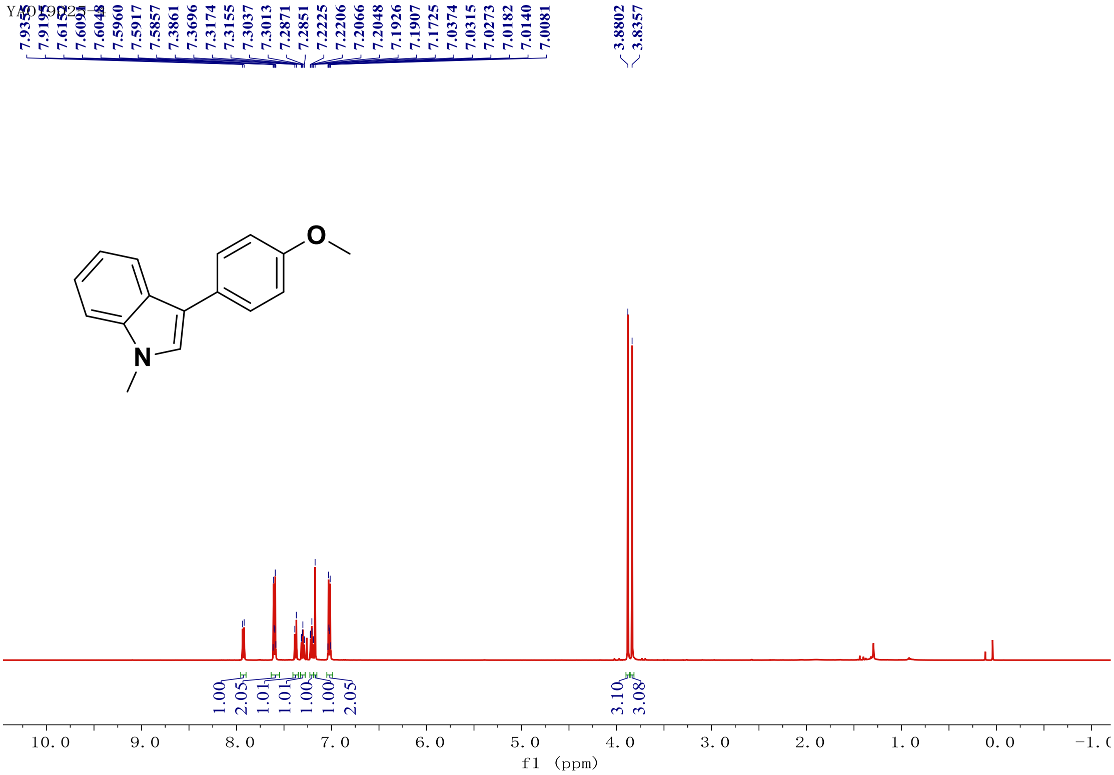

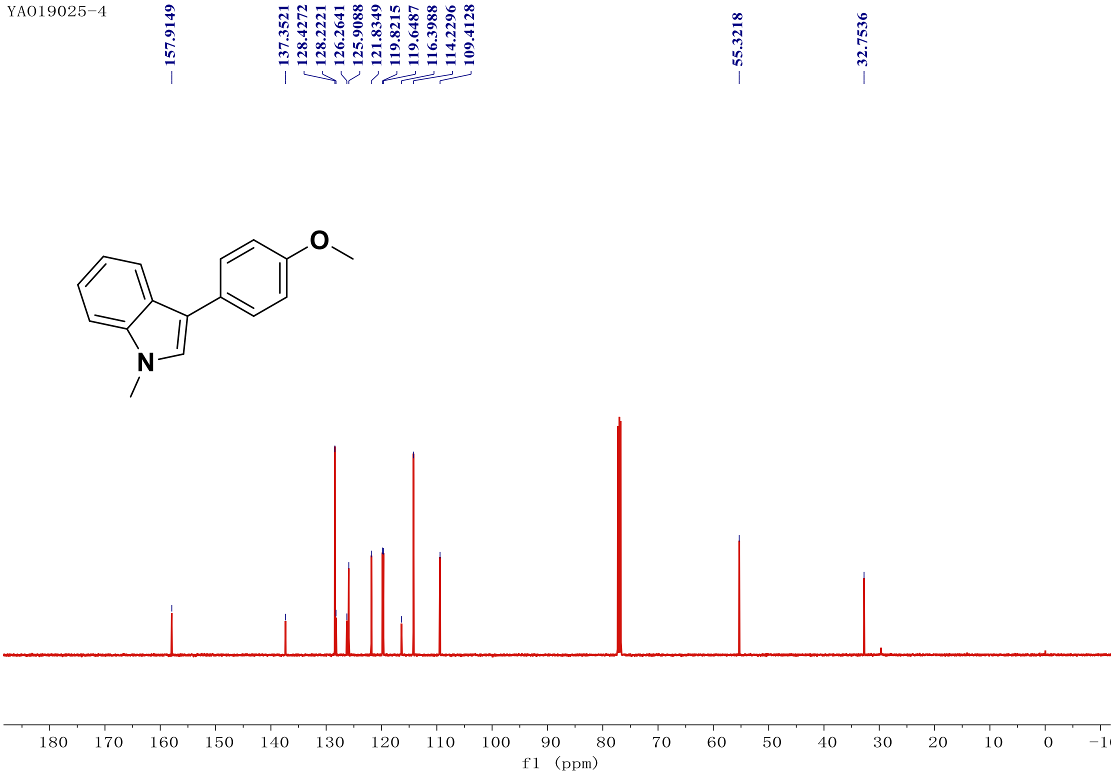

1-Methyl-3-(4-nitrophenyl)-1*H*-indole (**6**)

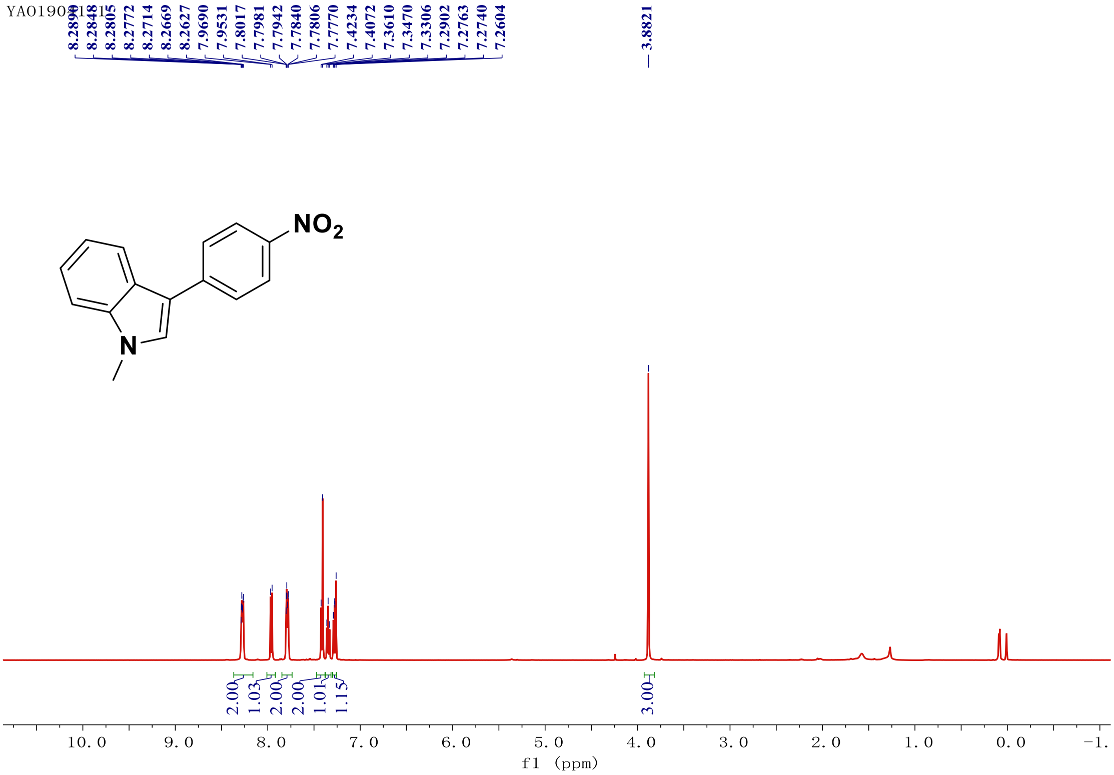

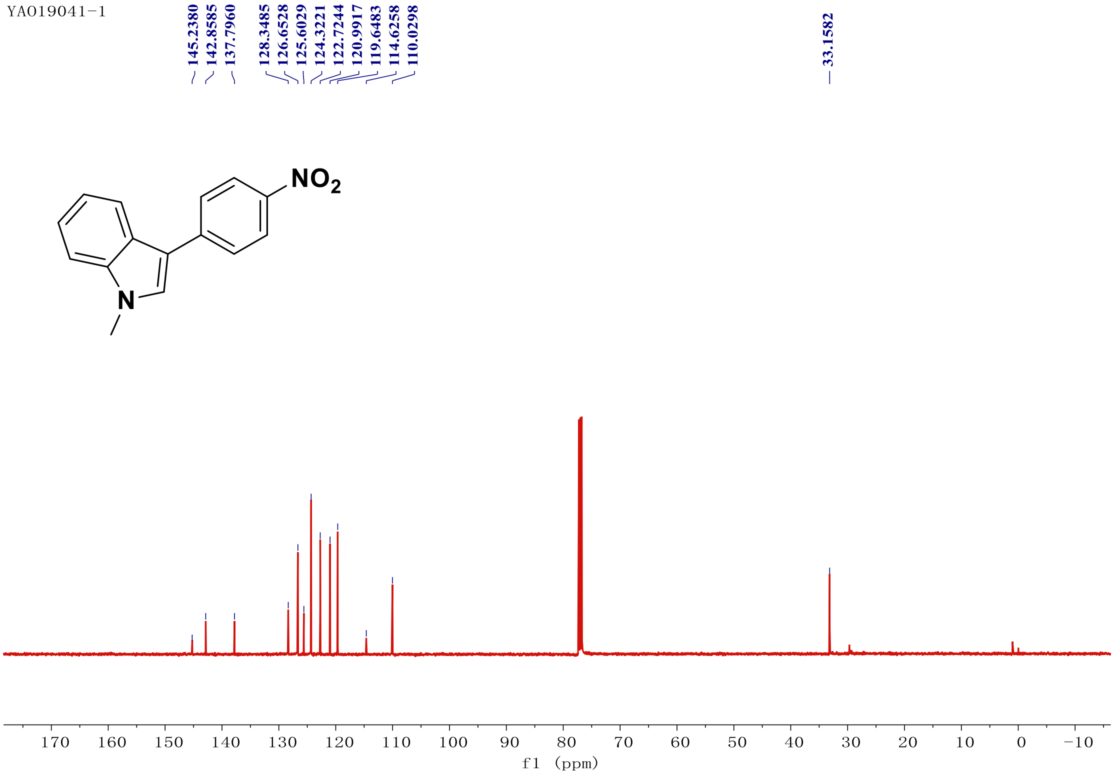

1-Methyl-3-(4-(trifluoromethyl)phenyl)-1*H*-indole (**7**)

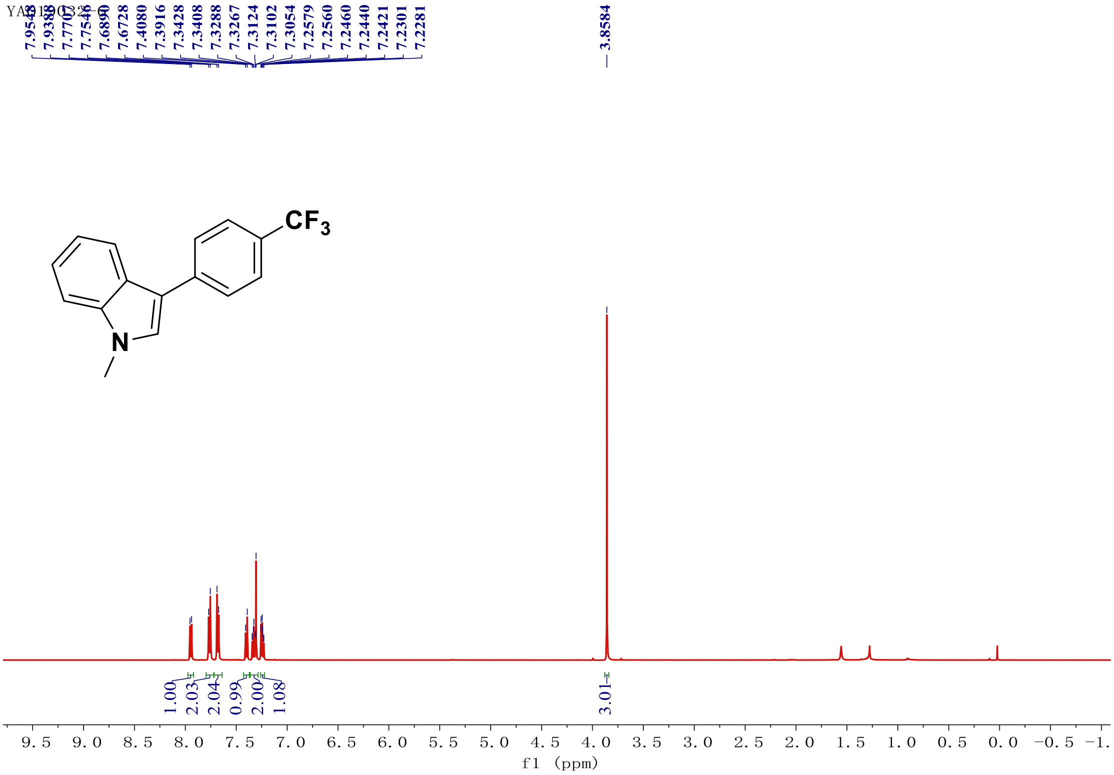

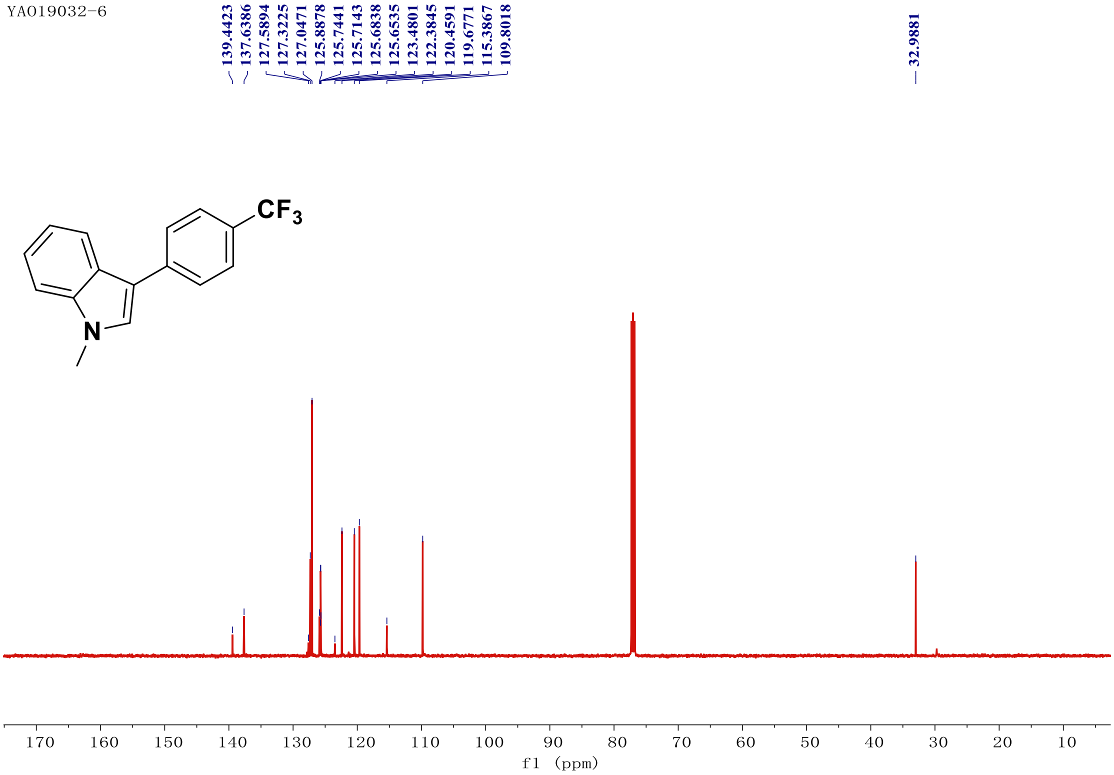

4-(1-Methyl-1*H*-indol-3-yl)benzonitrile (**8**)

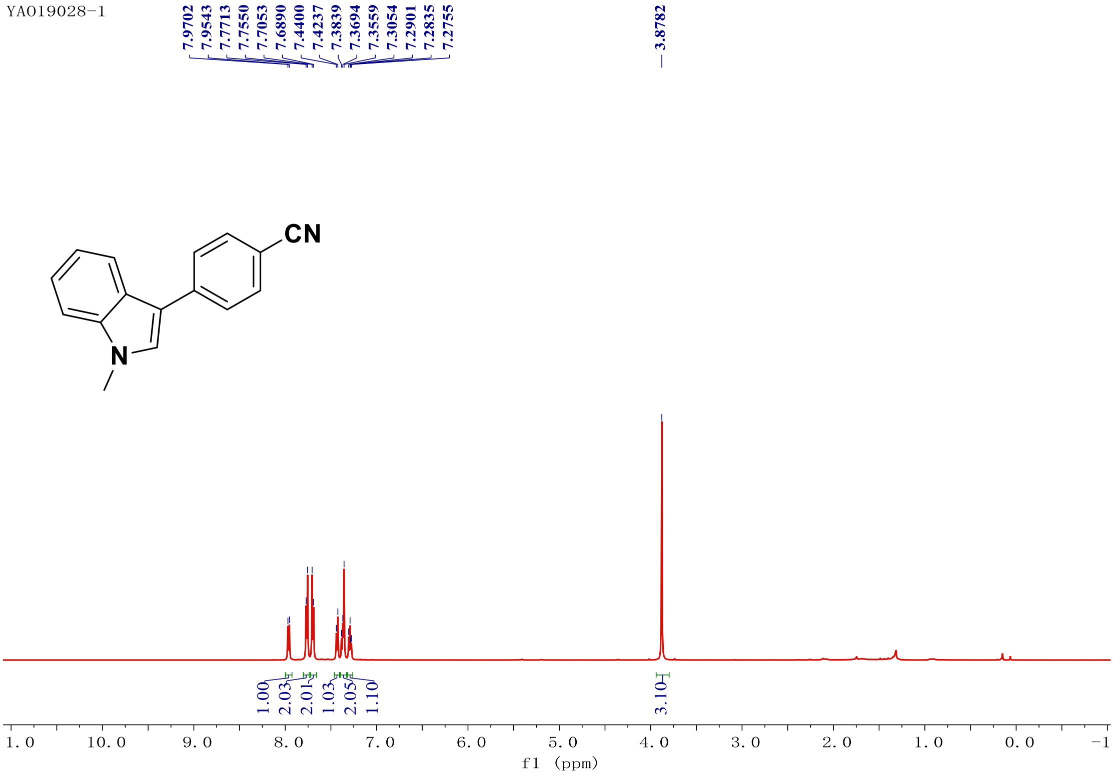

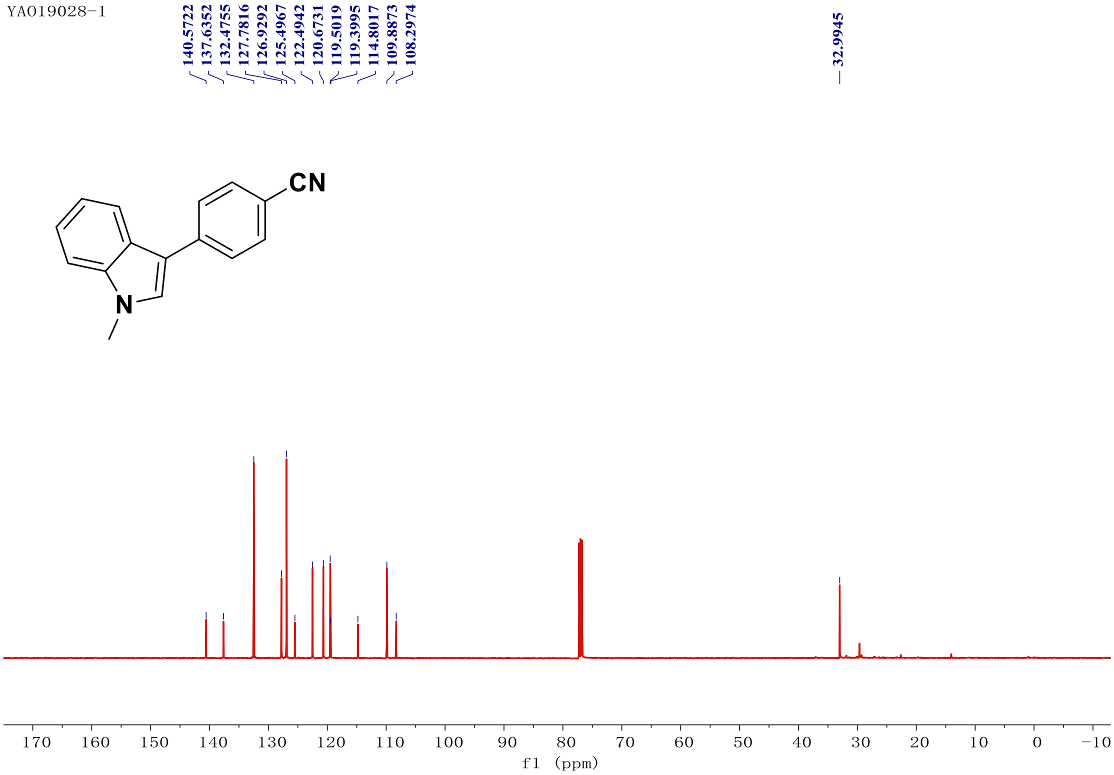

3-(4-Bromophenyl)-1-methyl-1*H*-indole (**9**)

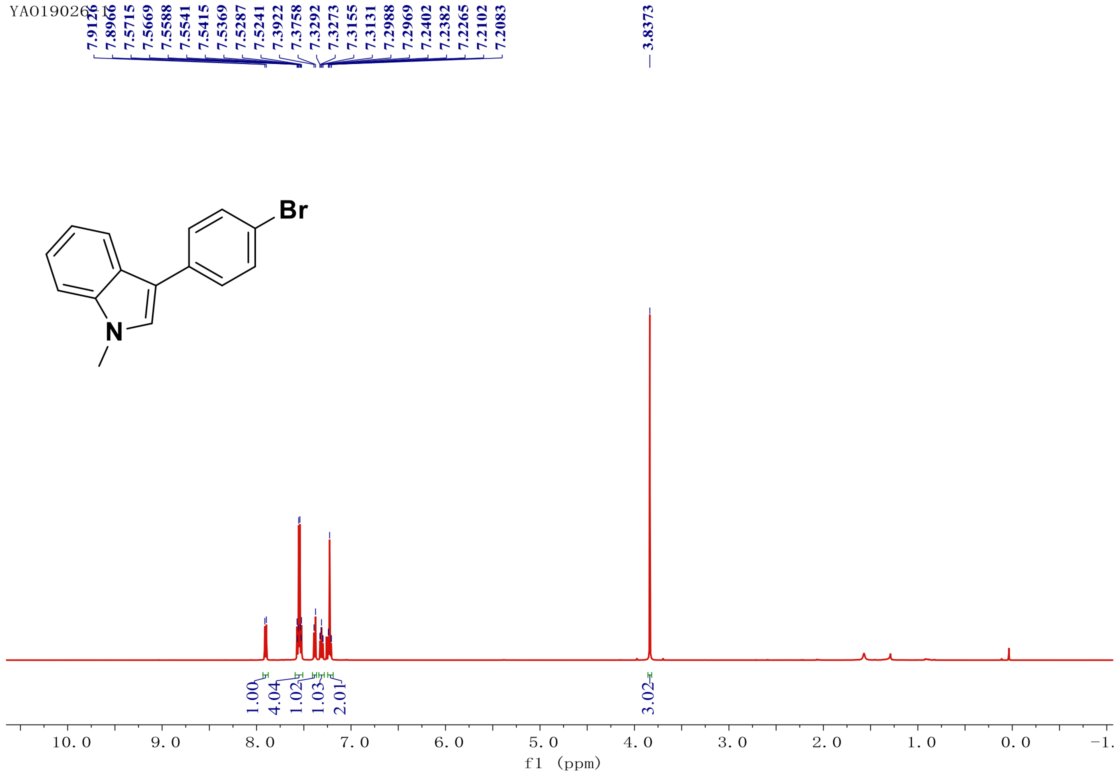

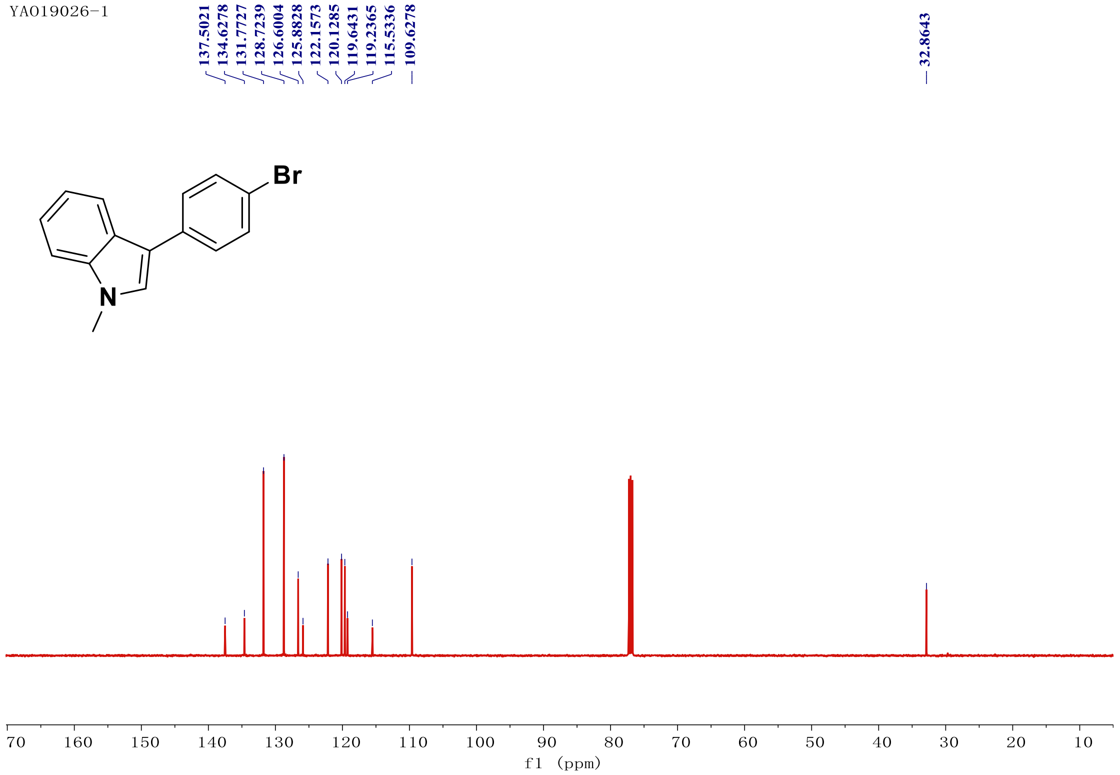

3-(4-Chlorophenyl)-1-methyl-1*H*-indole (**10**).

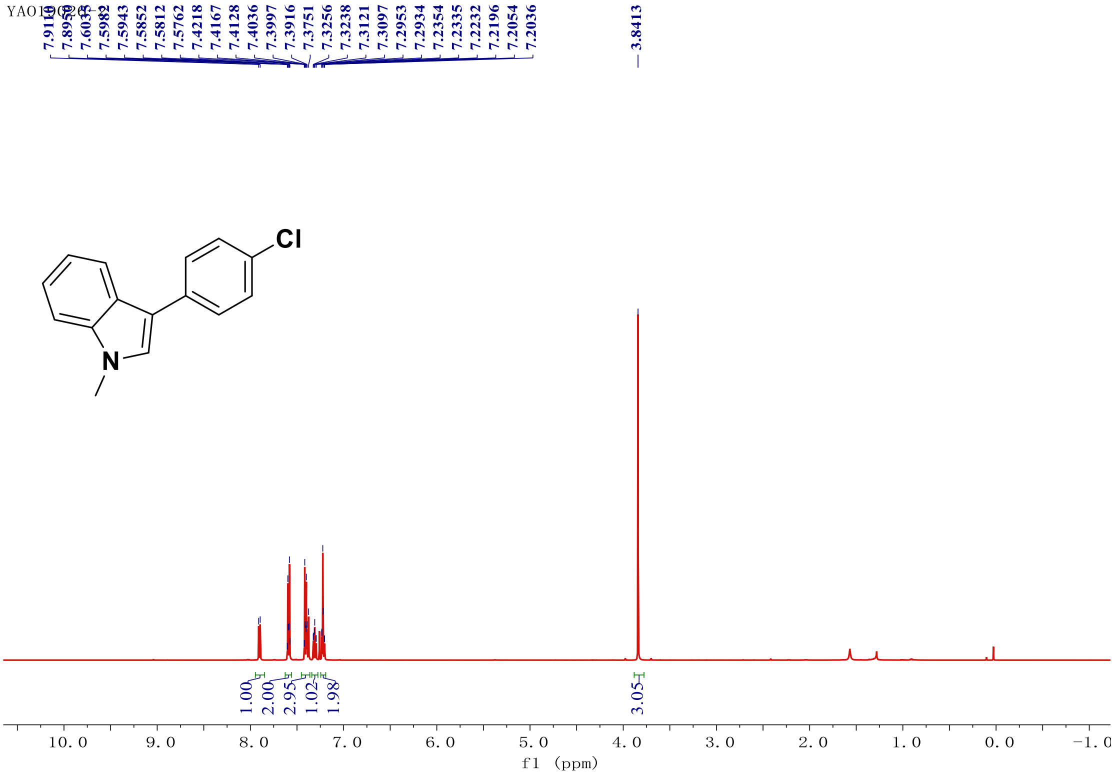

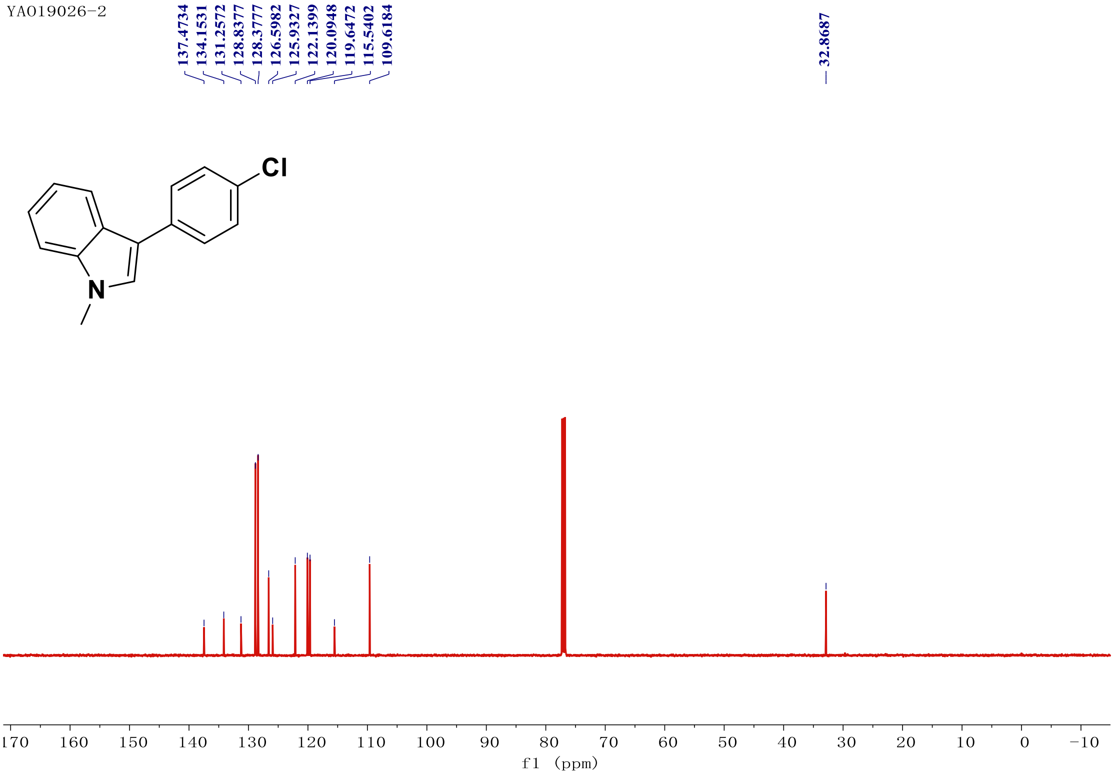

1-Methyl-3-(3-nitrophenyl)-1*H*-indole (**11**)

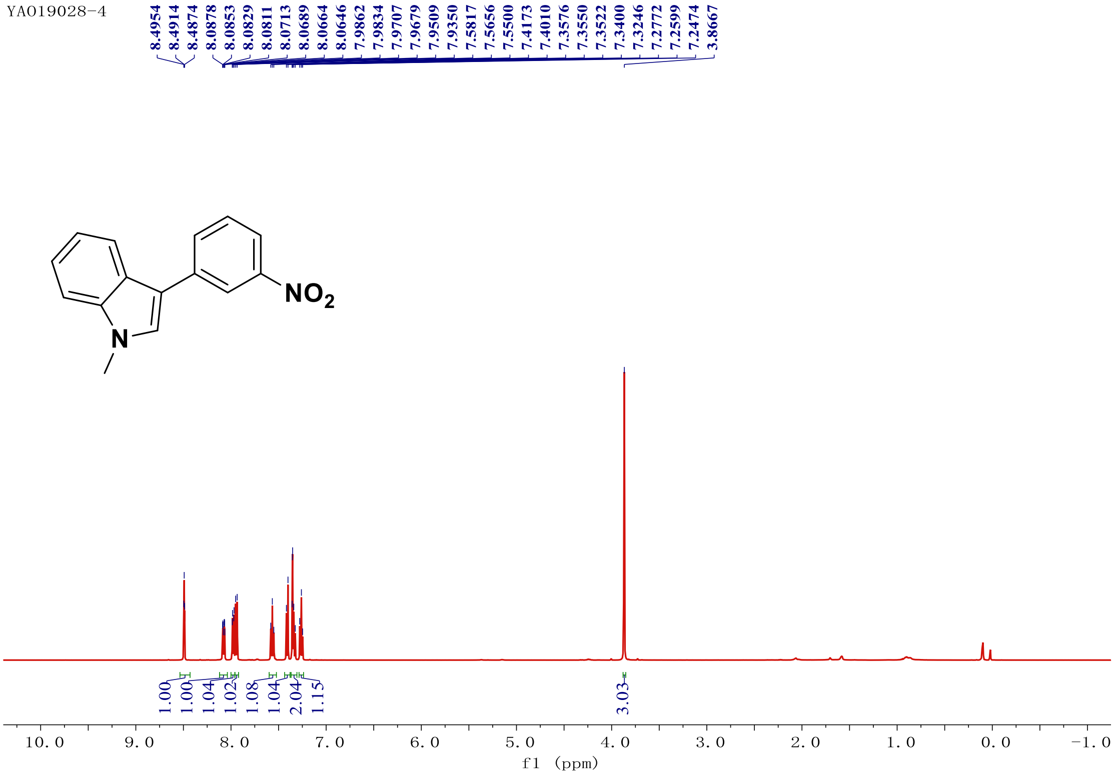

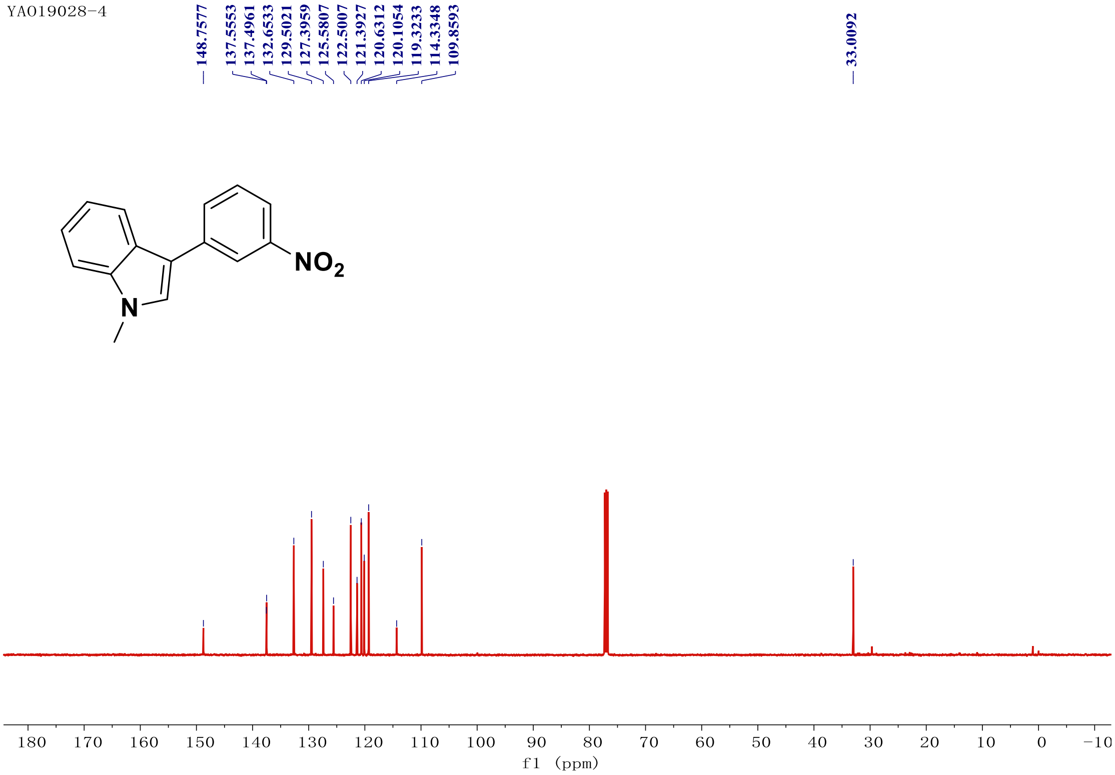

3-(1-Methyl-1*H*-indol-3-yl)benzonitrile (**12**)

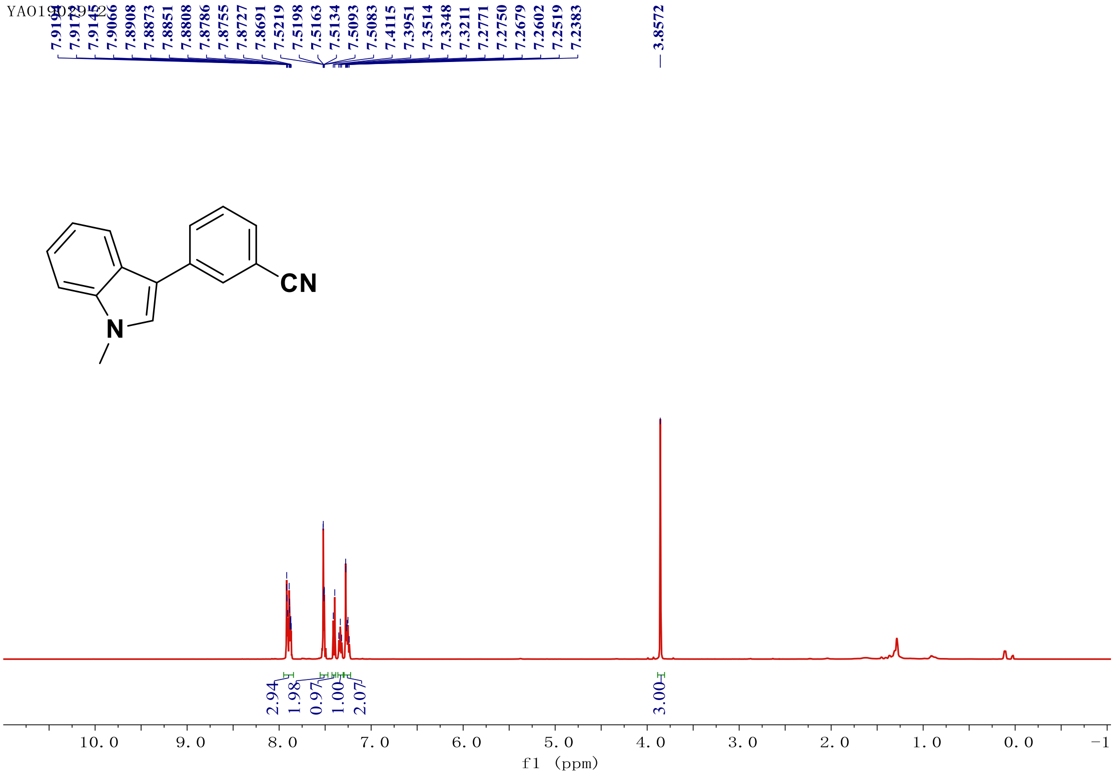

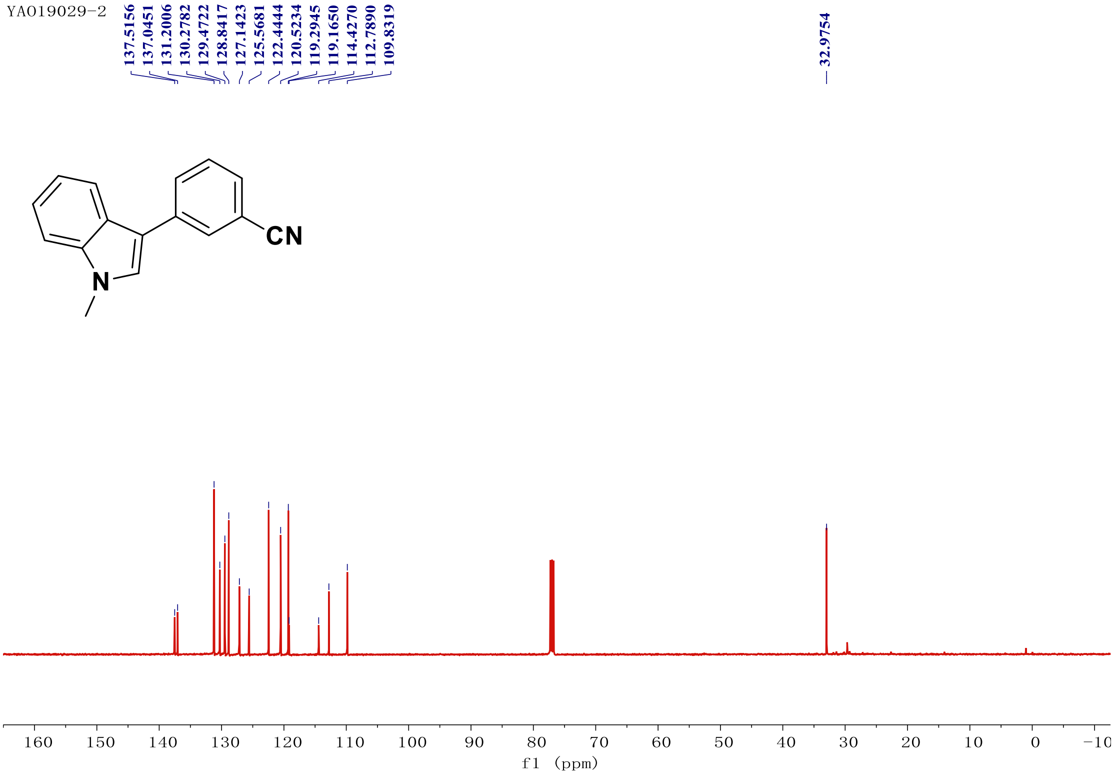

3-(3,4-Dimethoxyphenyl)-1-methyl-1*H*-indole (**13**)

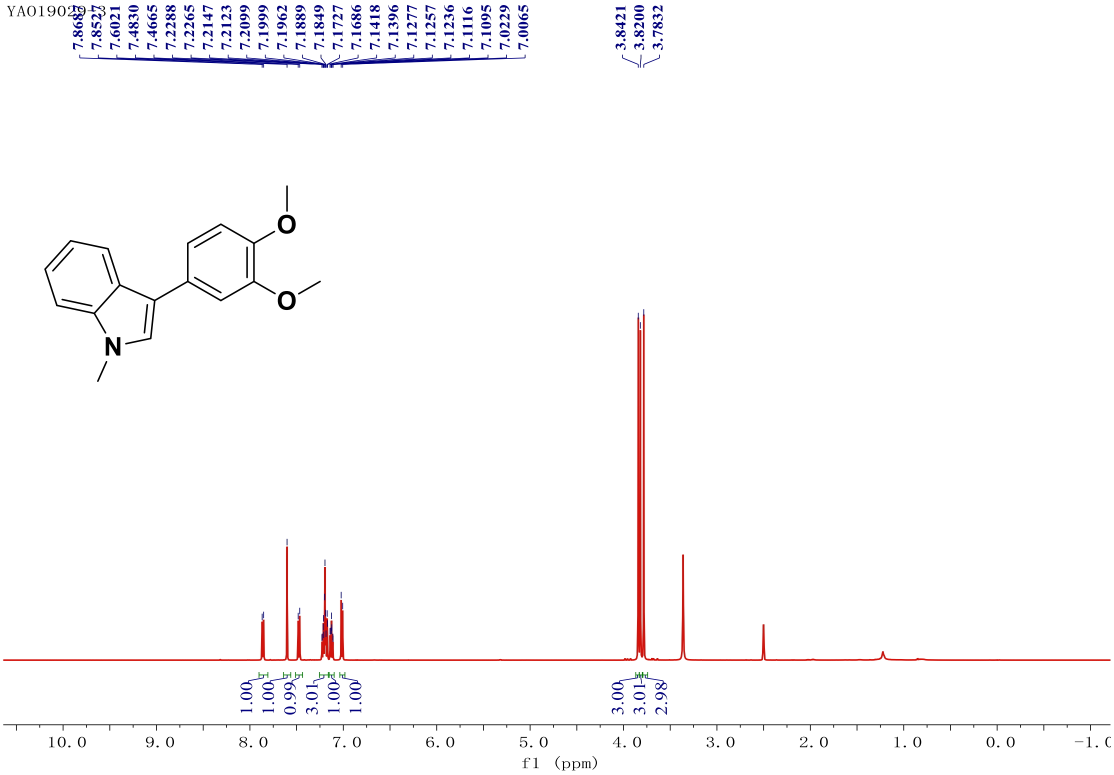

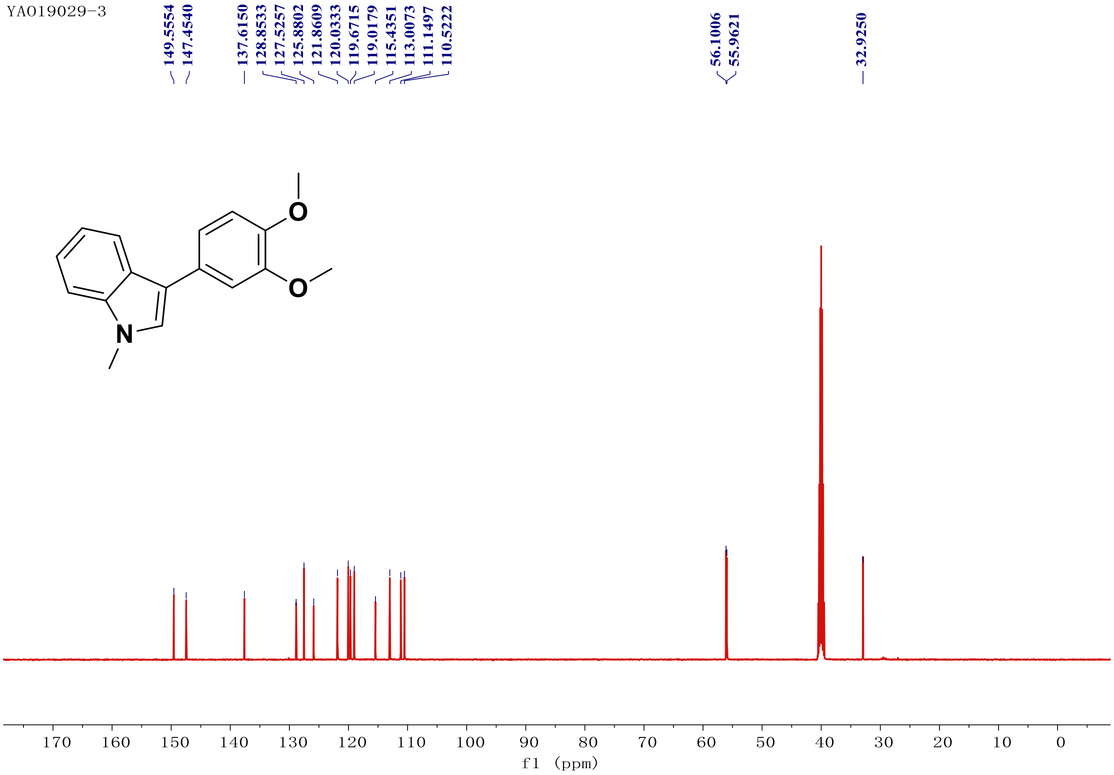

3-(3,4-Difluorophenyl)-1-methyl-1*H*-indole (**14**)

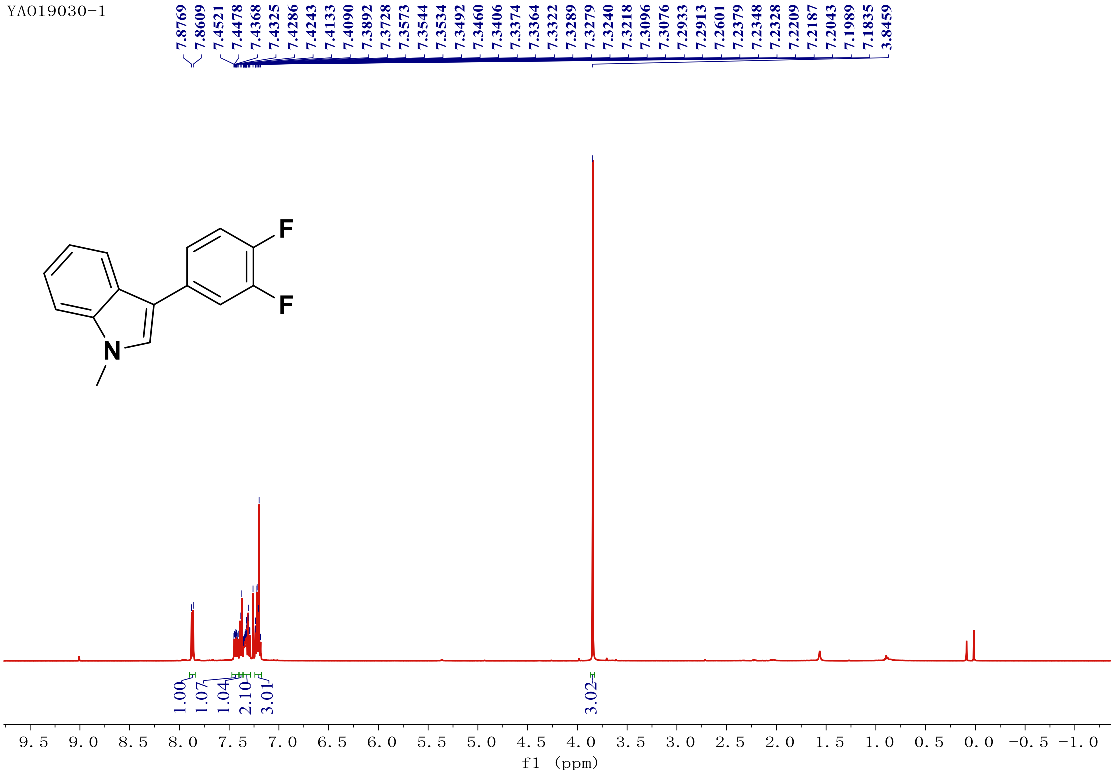

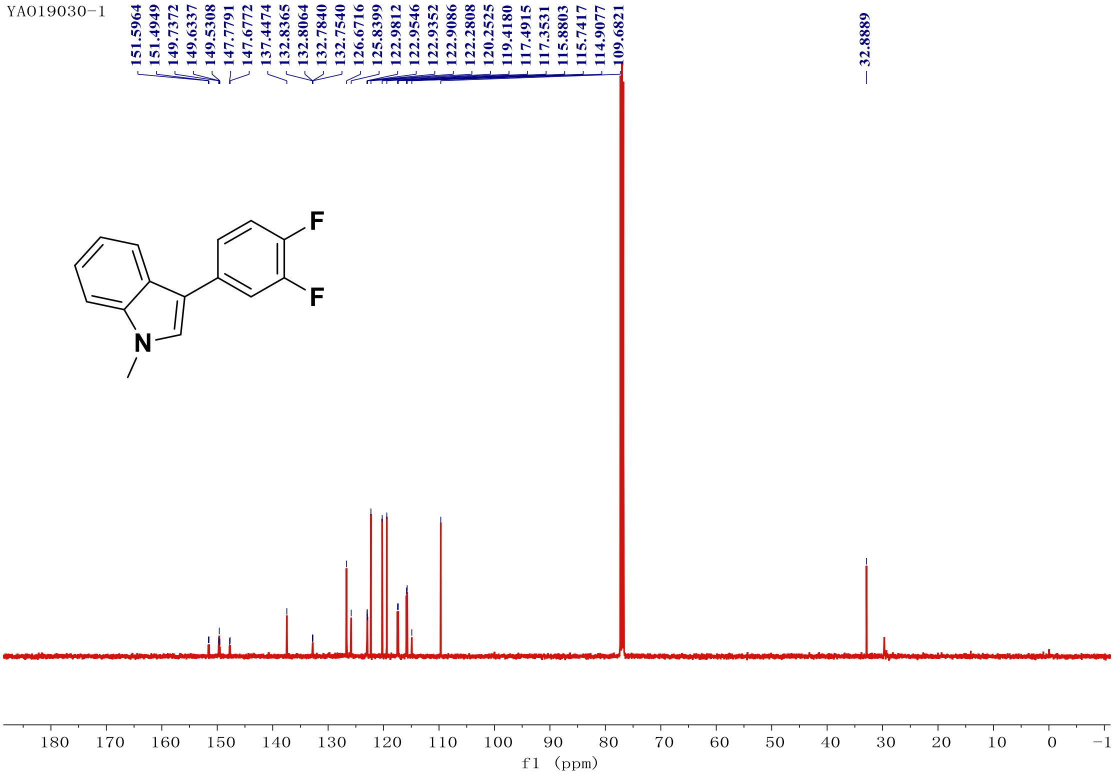

1-Methyl-3-(naphthalen-2-yl)-1*H*-indole (**15**)

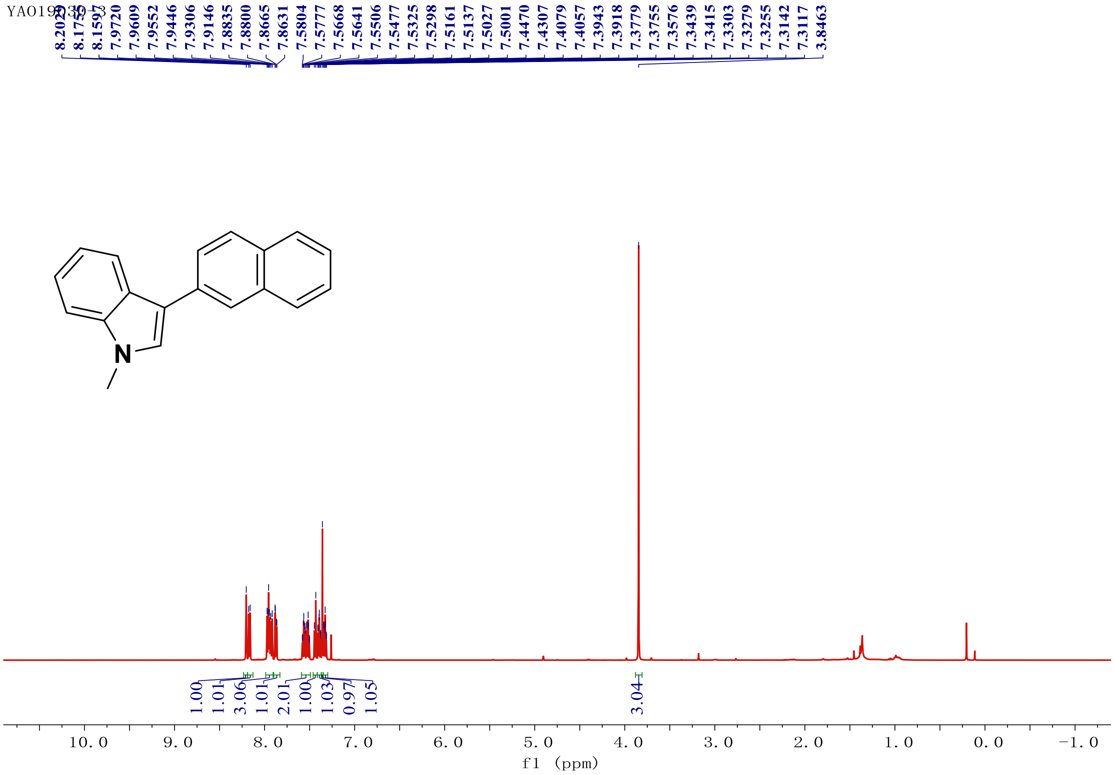

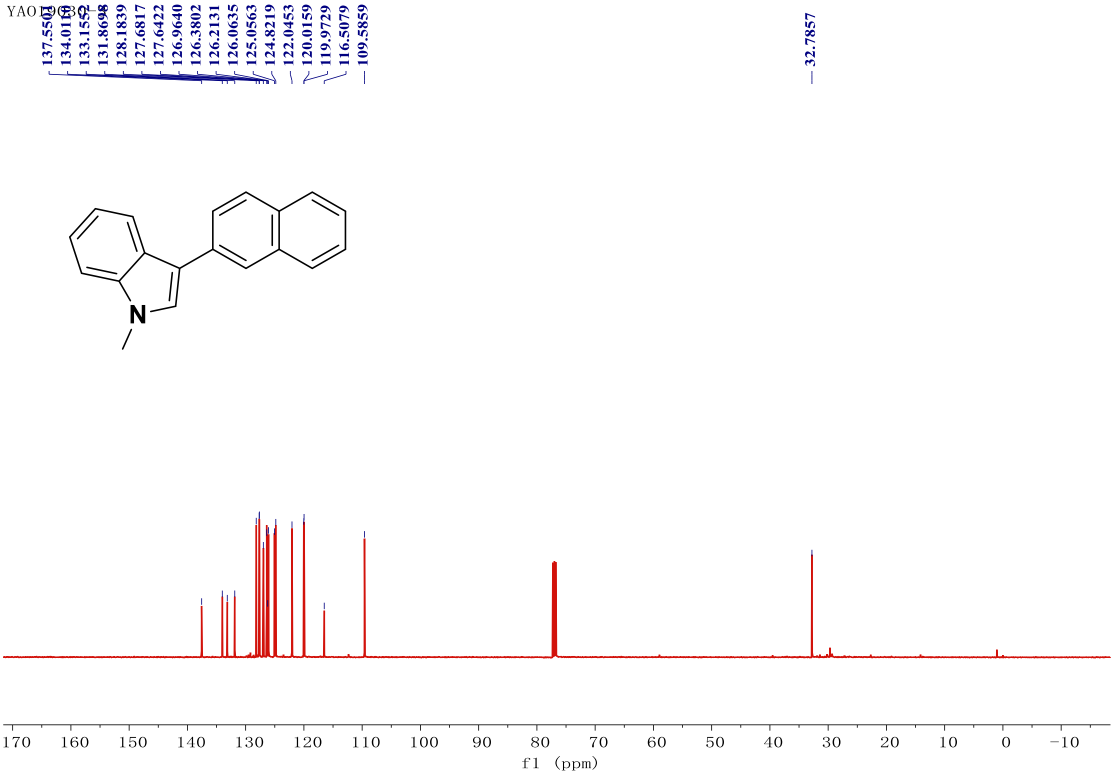

3-(3,4-Dihydro-2H-benzo[b][1,4]dioxepin-7-yl)-1-methyl-1*H*-indole (**16**)

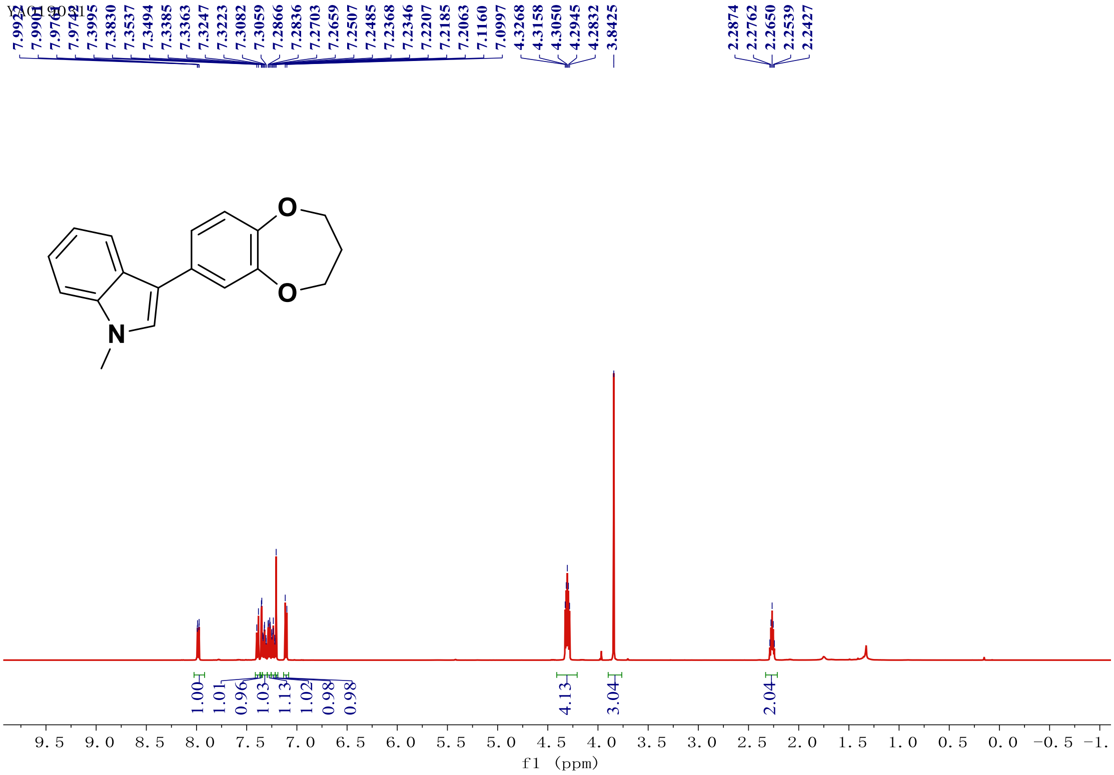

YA019031

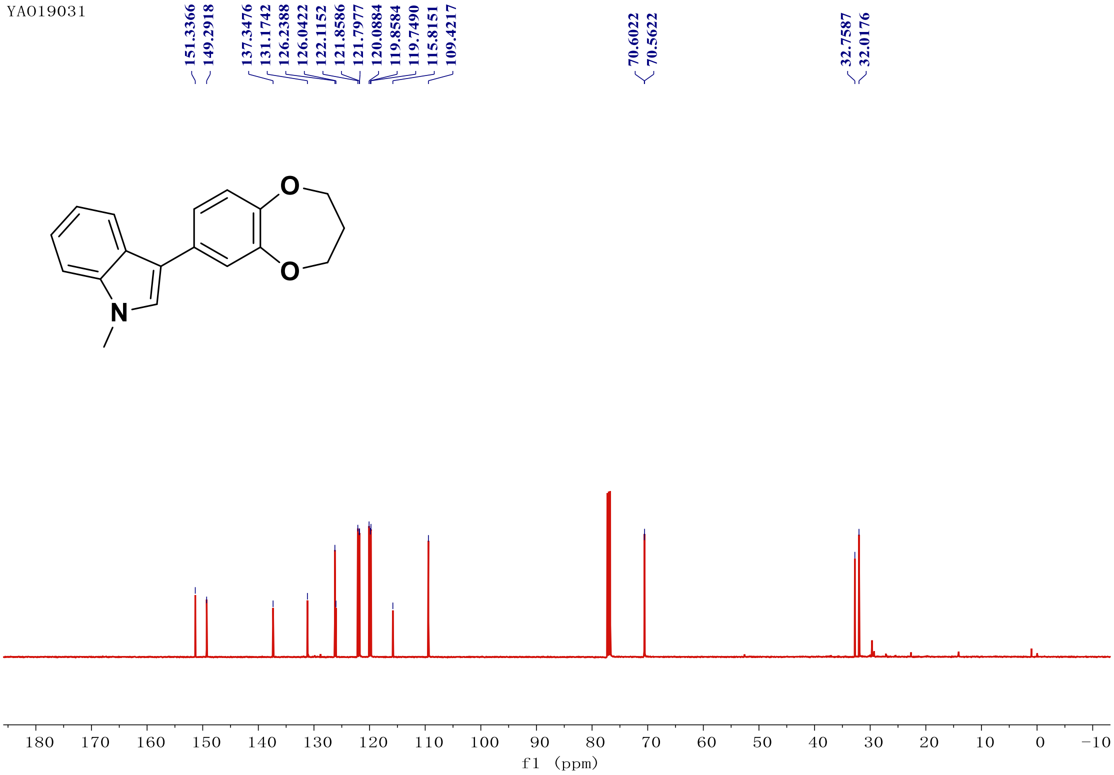

3-(Benzofuran-2-yl)-1-methyl-1*H*-indole (**17**)

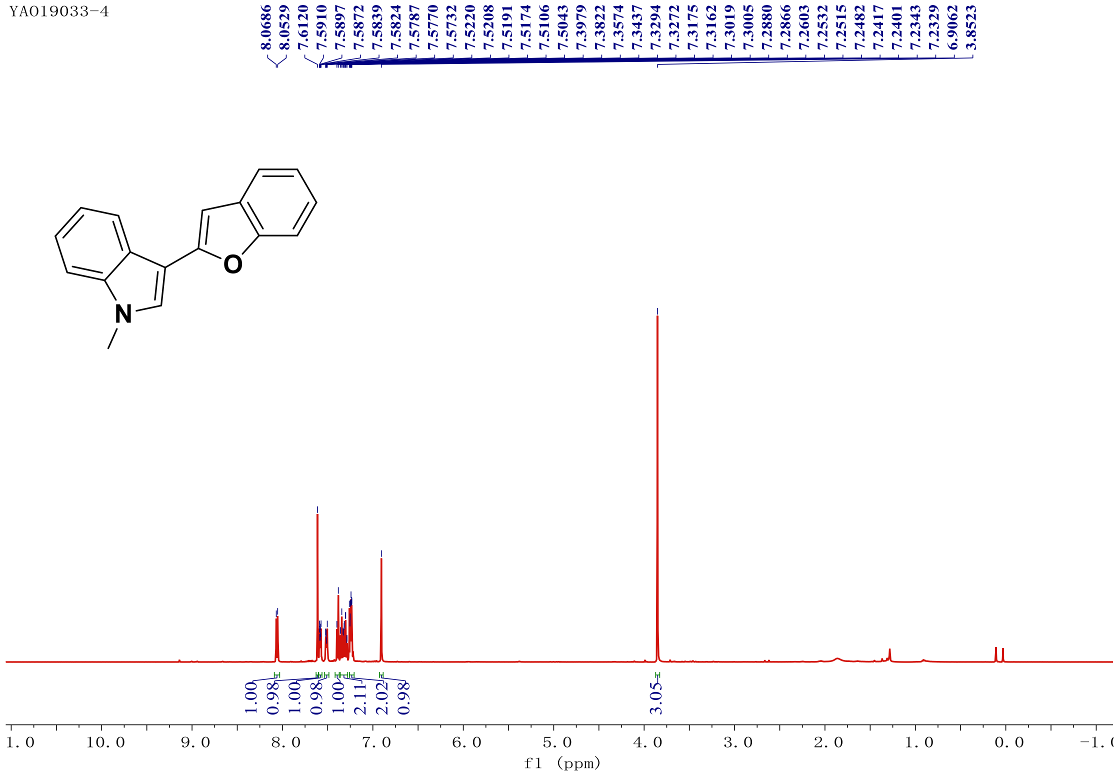

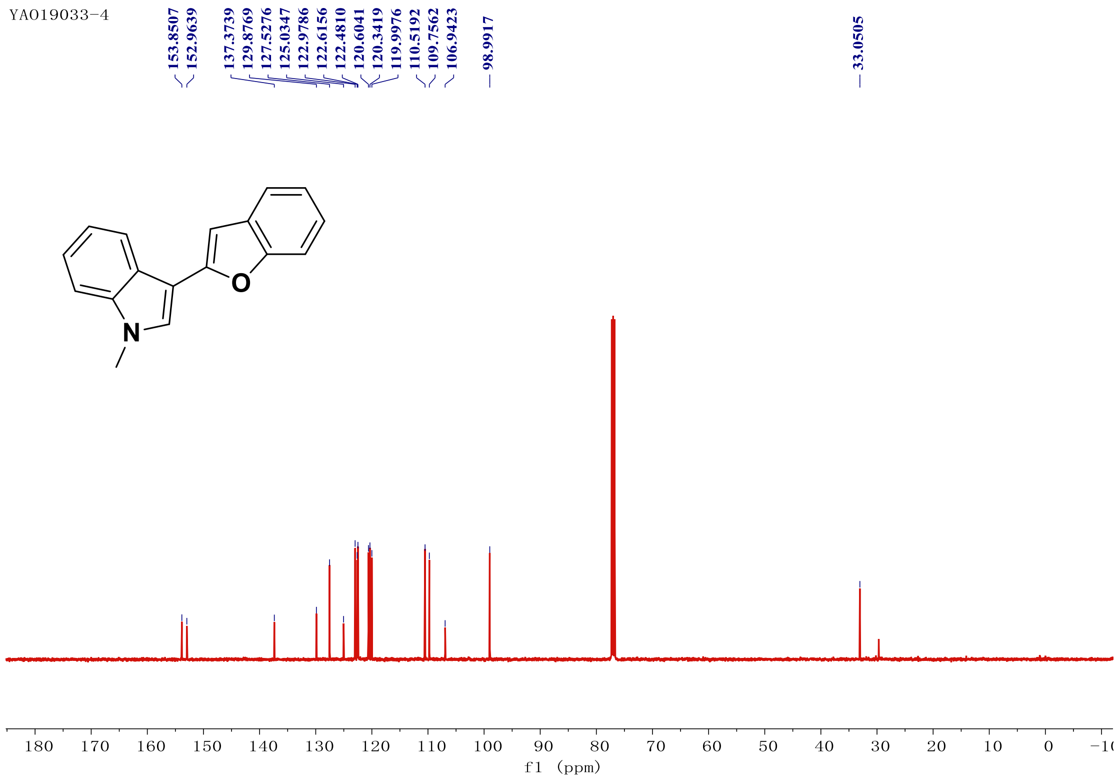

Ethyl 1-methyl-1*H*-indole-3-carboxylate (**18**)

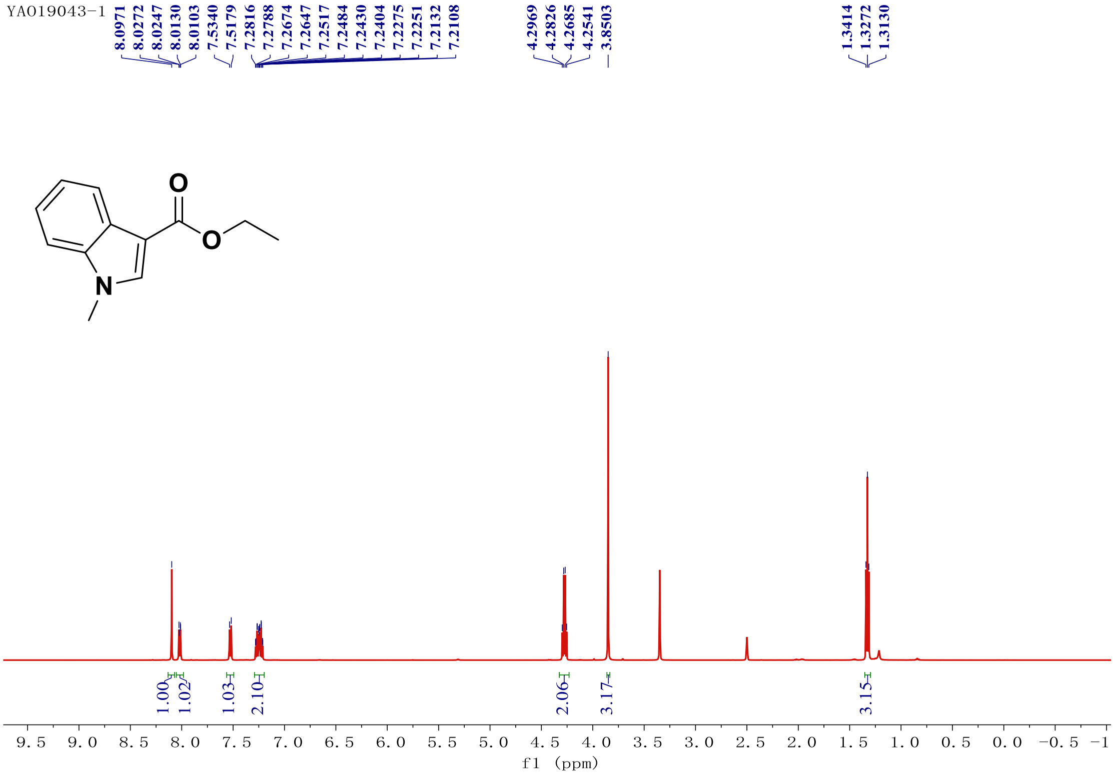

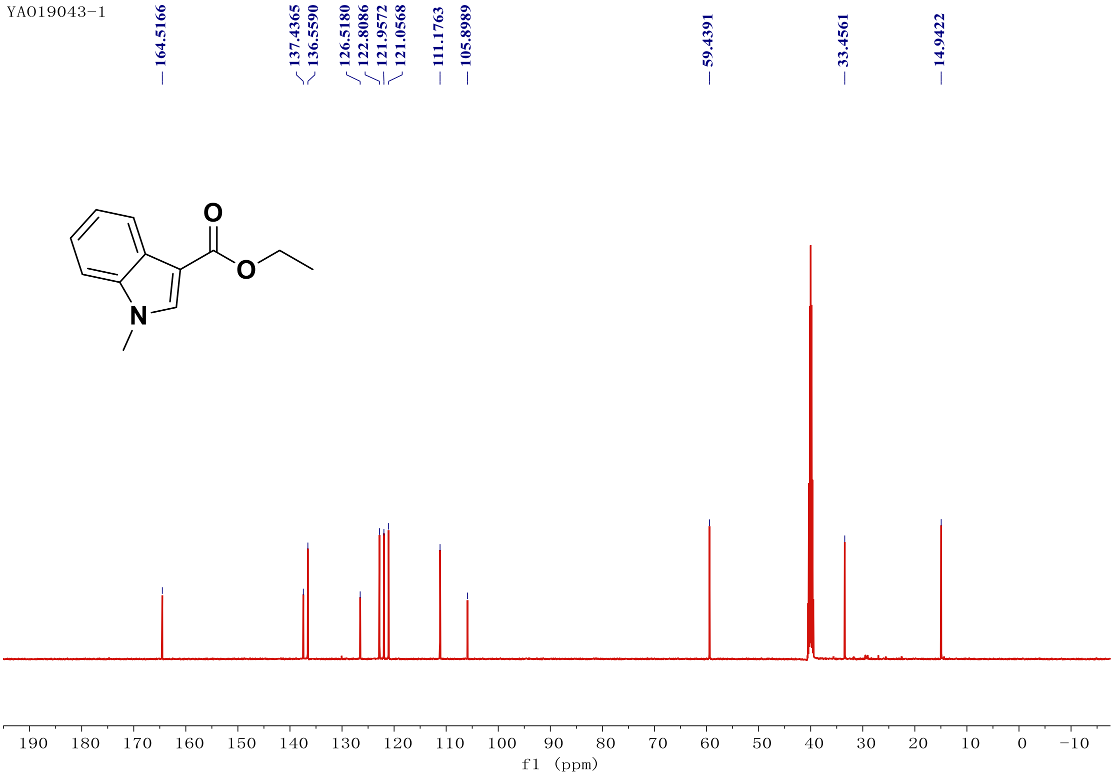

Ethyl 2-(1-methyl-1*H*-indol-3-yl)acetate (**19**)

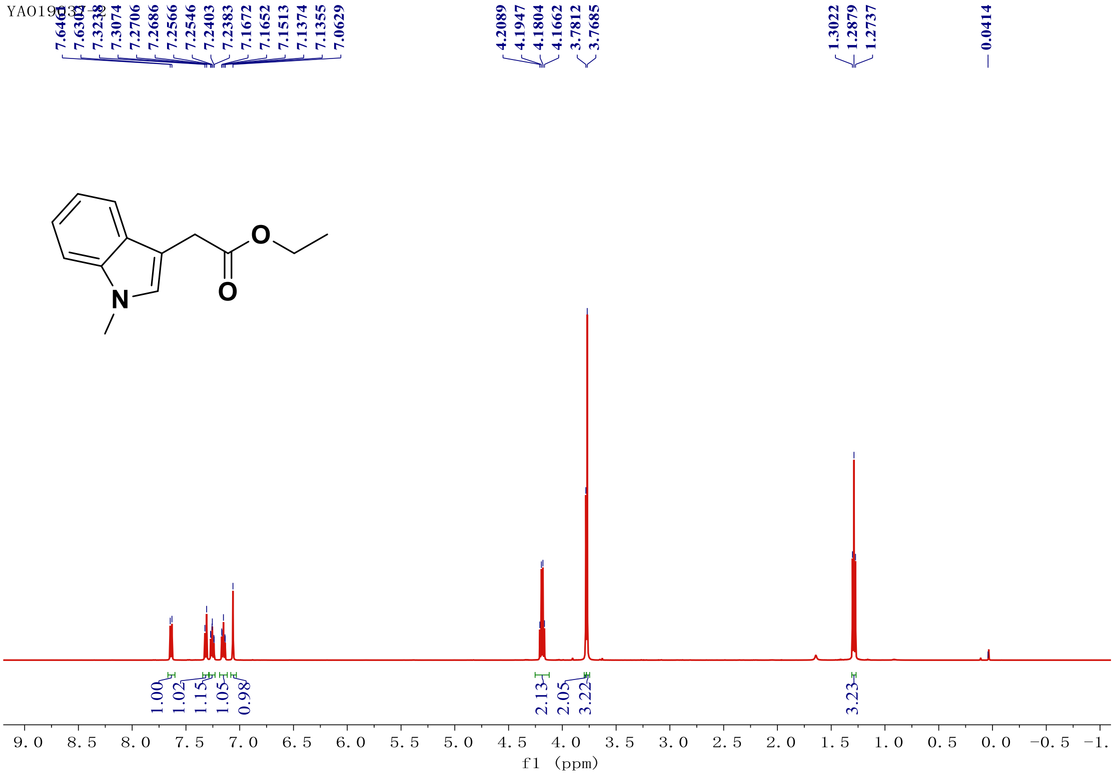

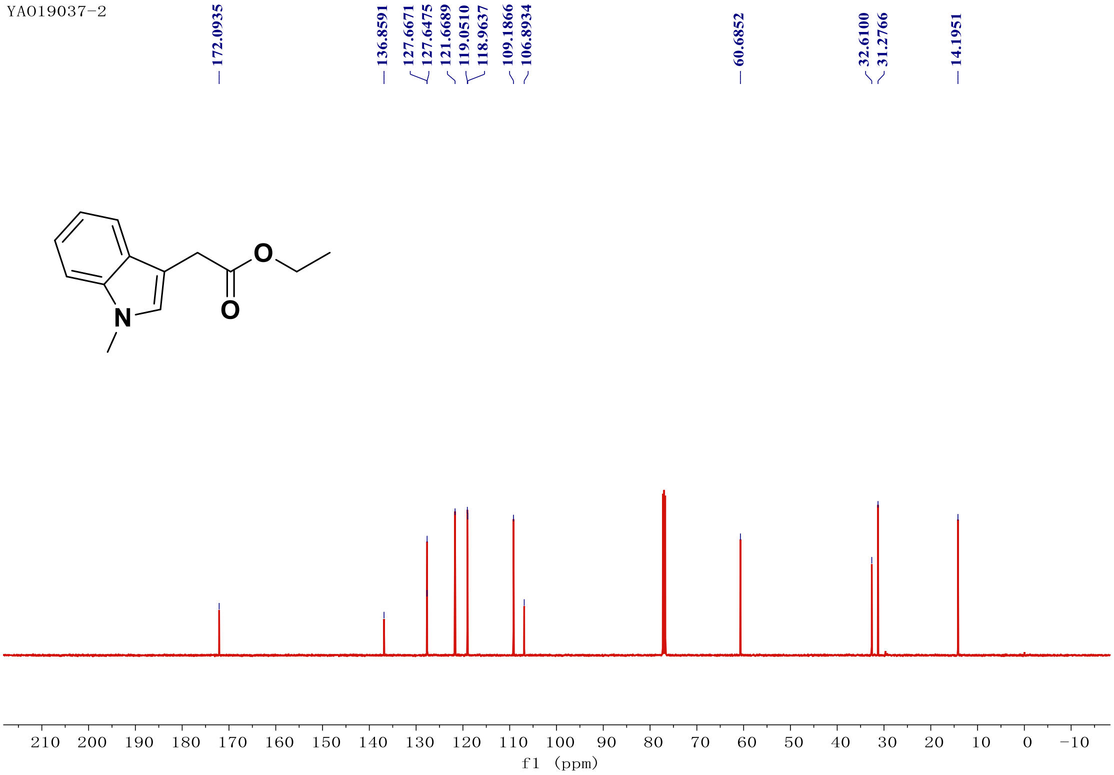

5-Methoxy-1-methyl-3-phenyl-1*H*-indole (**20**)

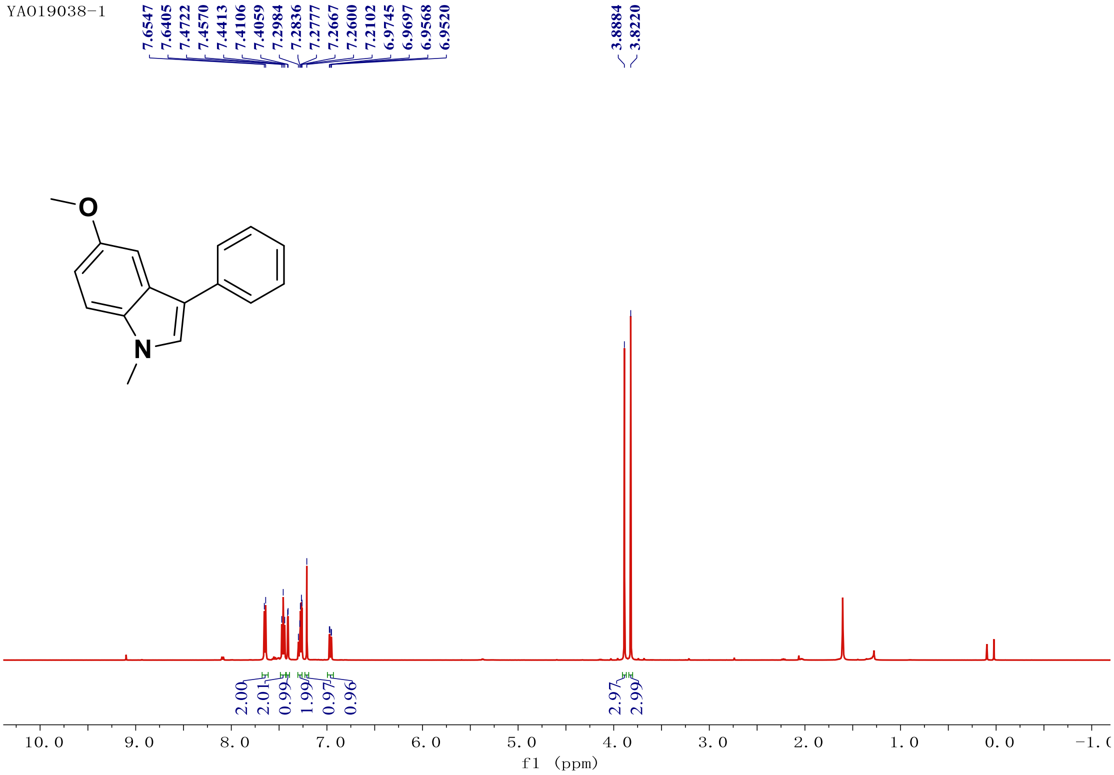

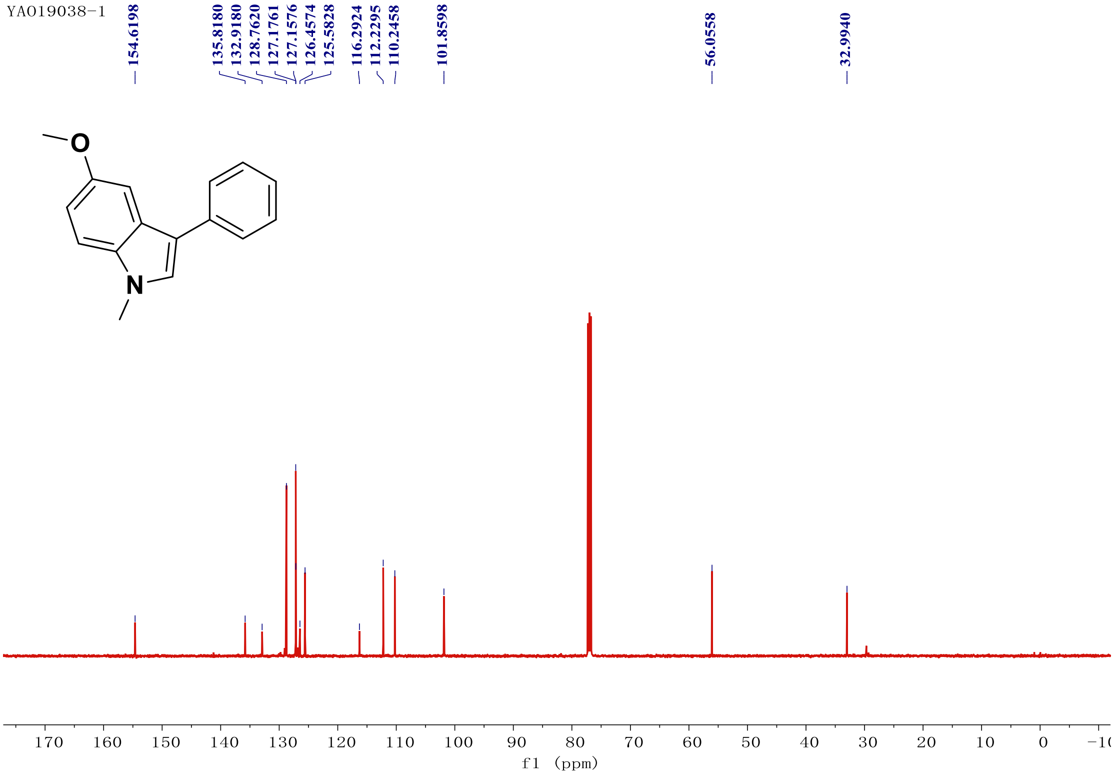

5-Bromo-1-methyl-3-phenyl-1*H*-indole (**21**)

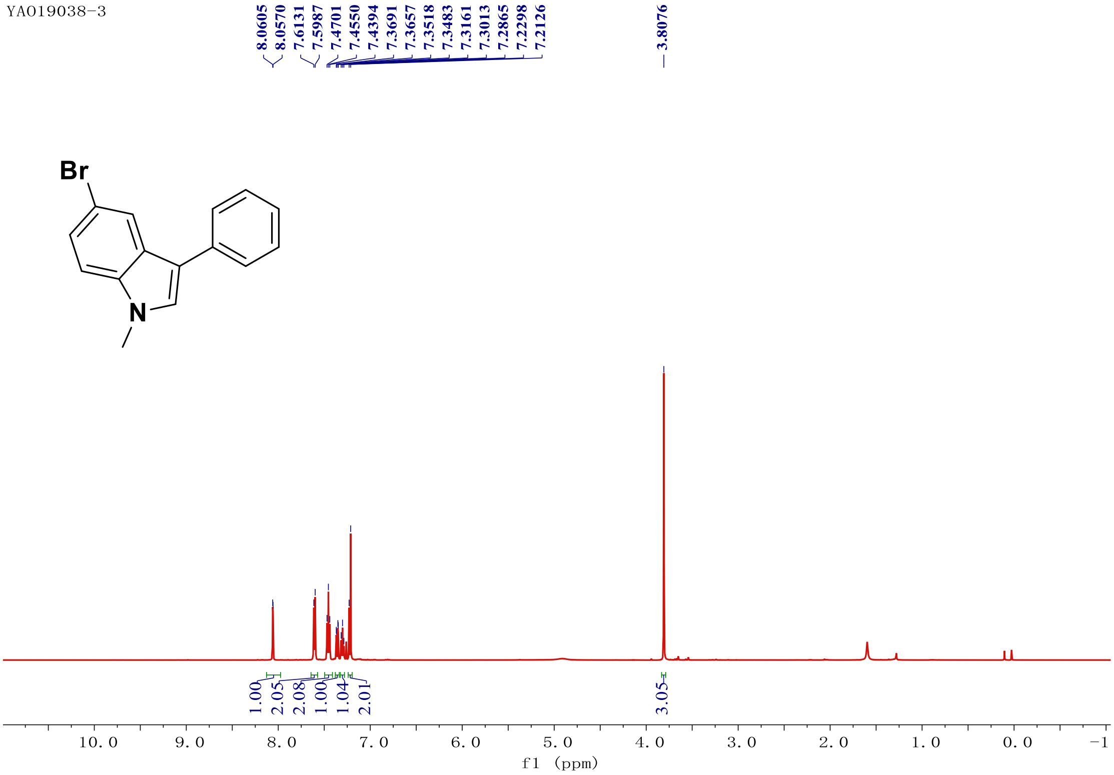

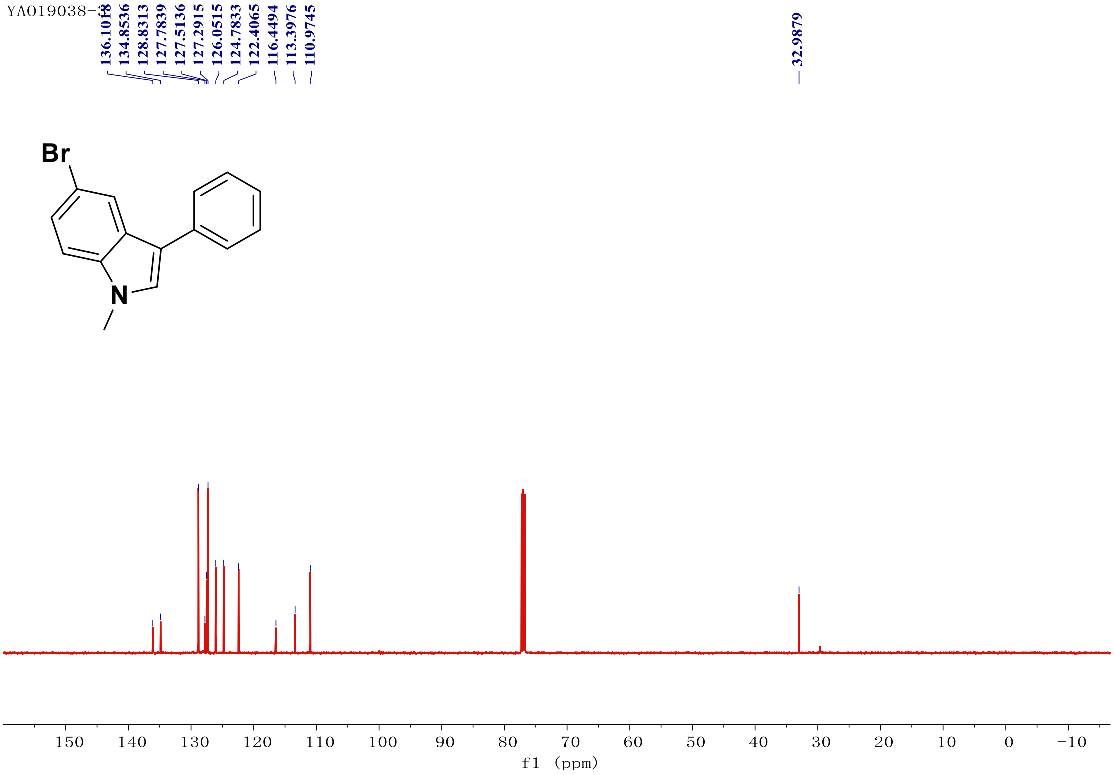

5-Chloro-1-methyl-3-phenyl-1*H*-indole (**22**)

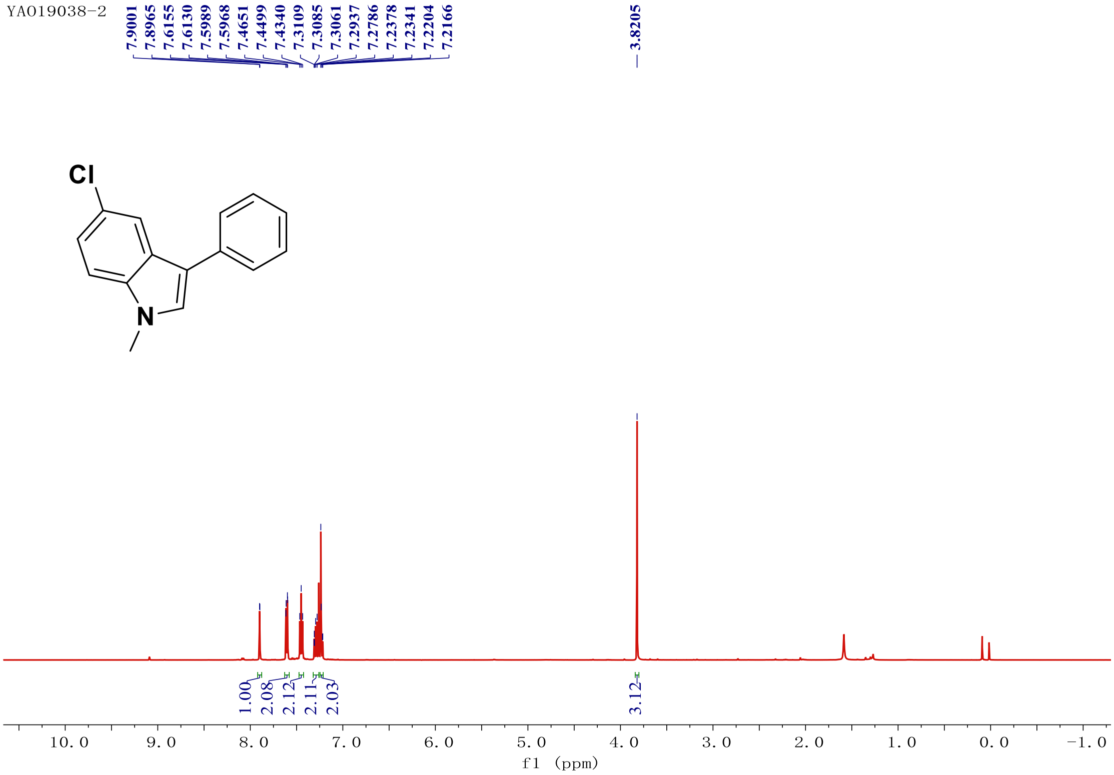

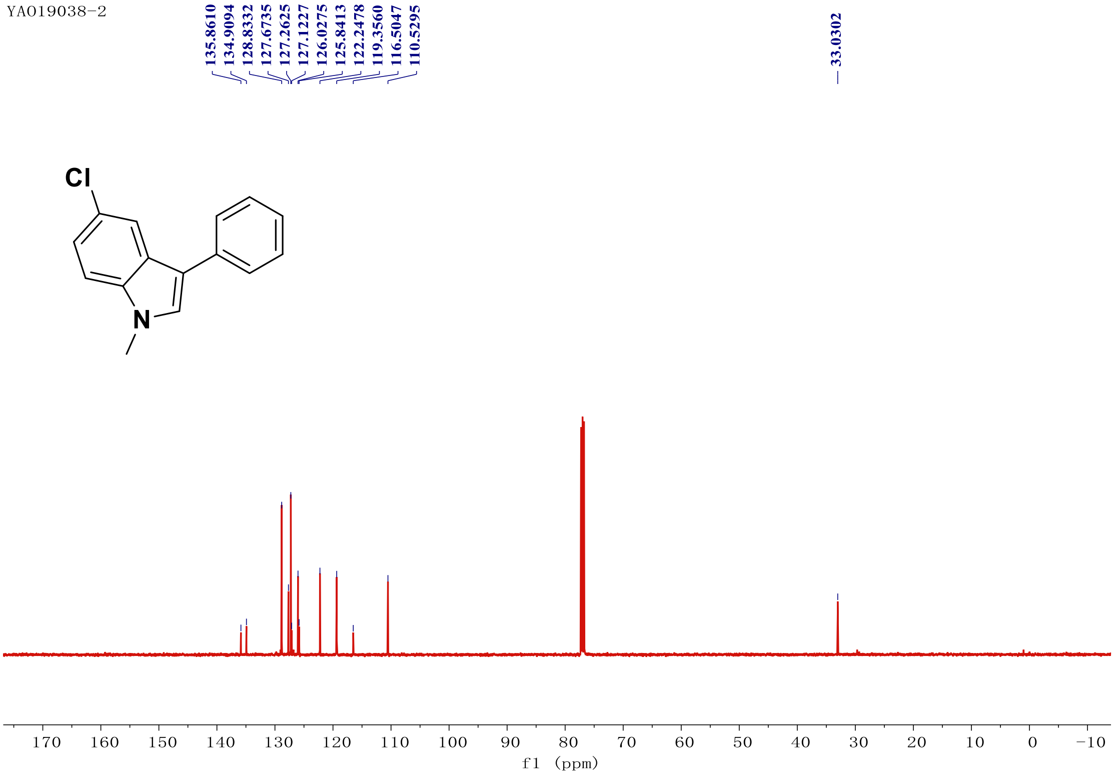

1-Benzyl-3-phenyl-1*H*-indole (**24**)

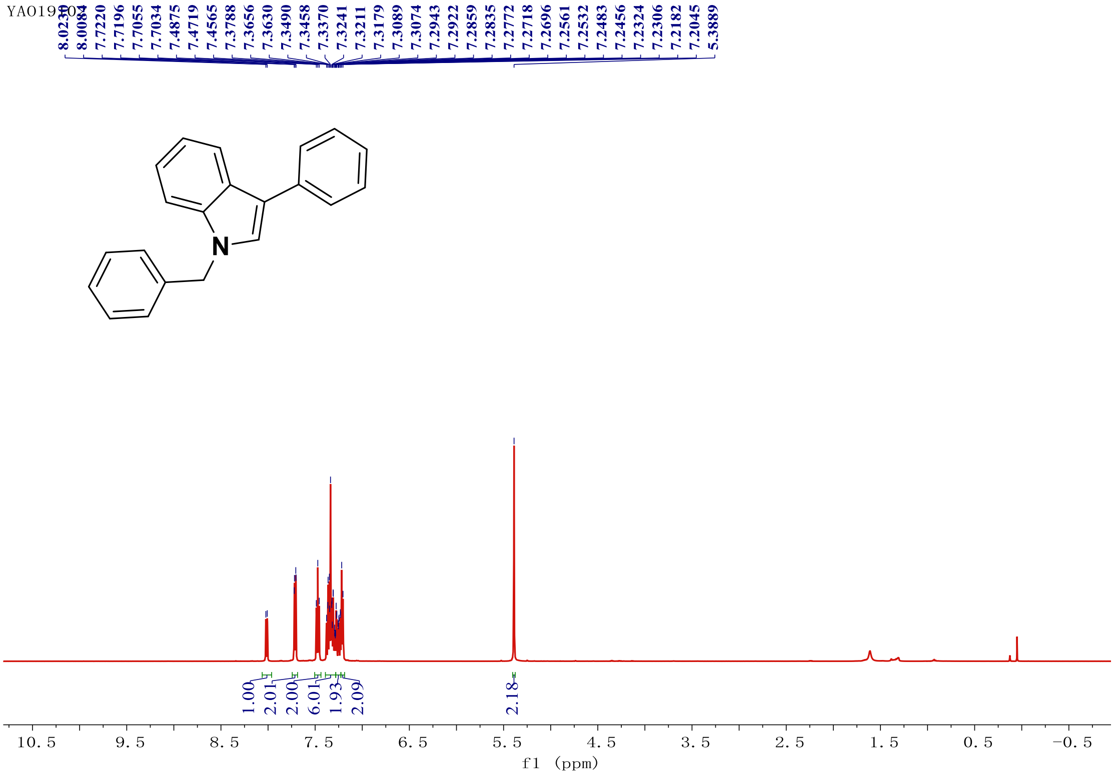

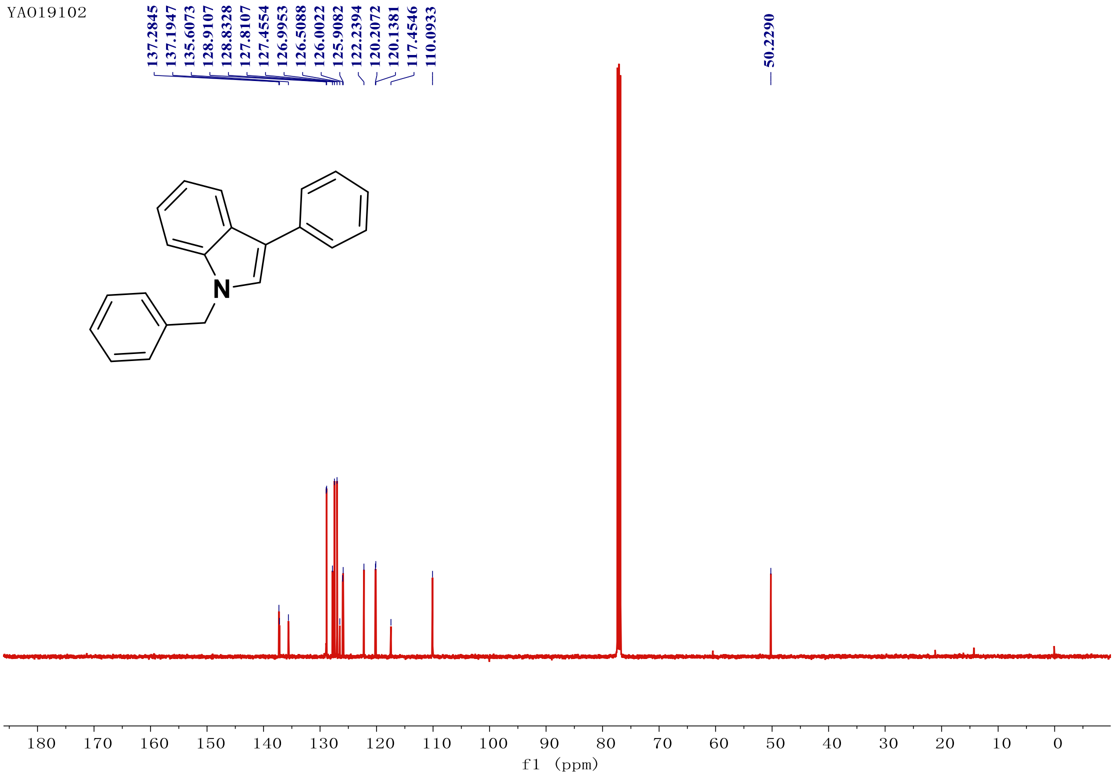

1-(4-Methoxyphenyl)-5,6-dihydro-4*H*-pyrrolo[3,2,1-*ij*]quinoline (**25**)

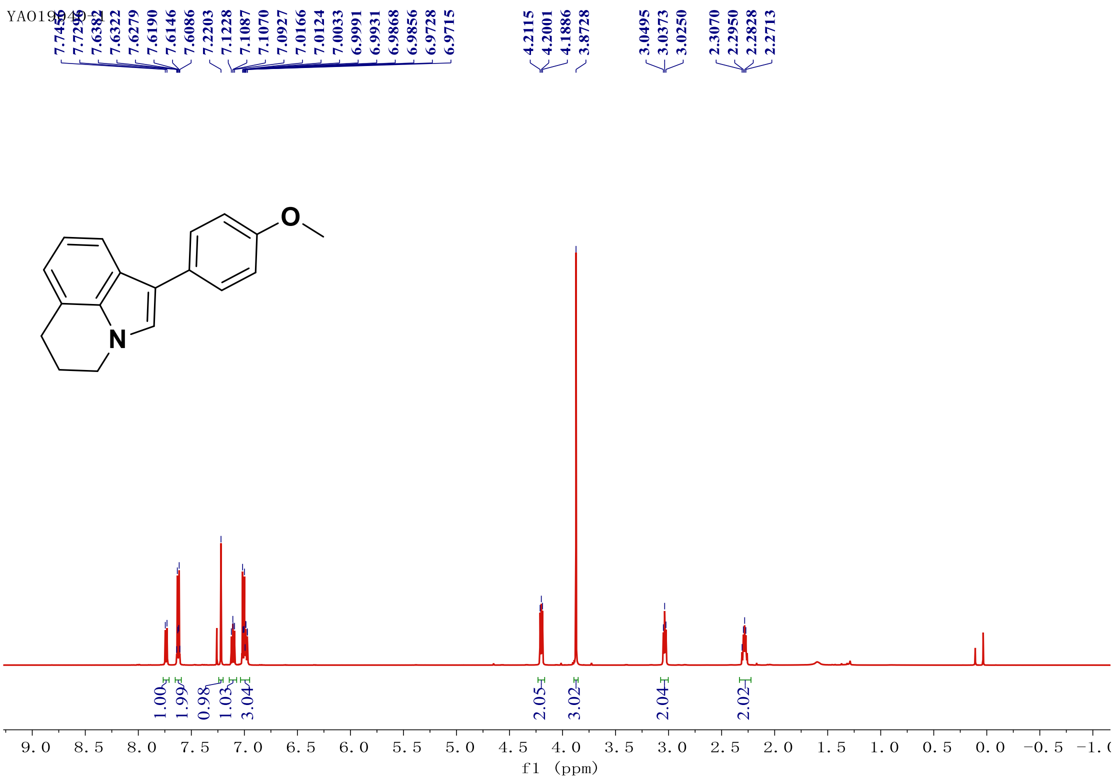

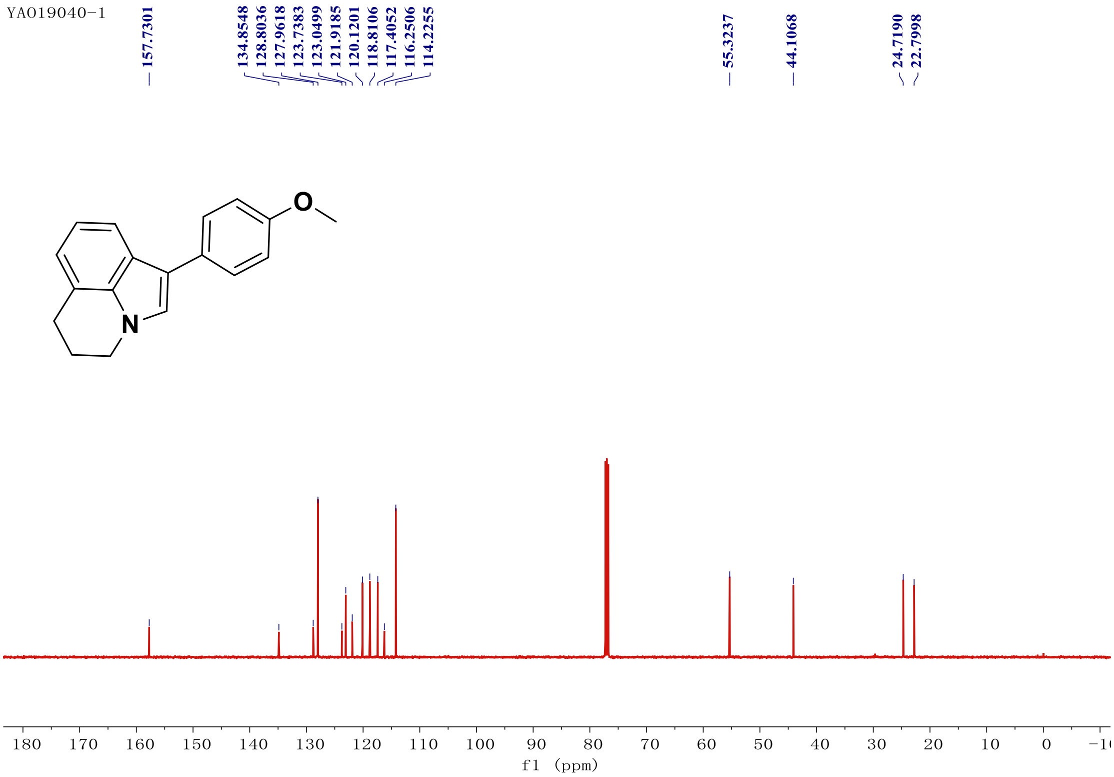

1-[4-(Trifluoromethyl)phenyl]-5,6-dihydro-4*H*-pyrrolo[3,2,1-*ij*]quinoline (**26**)

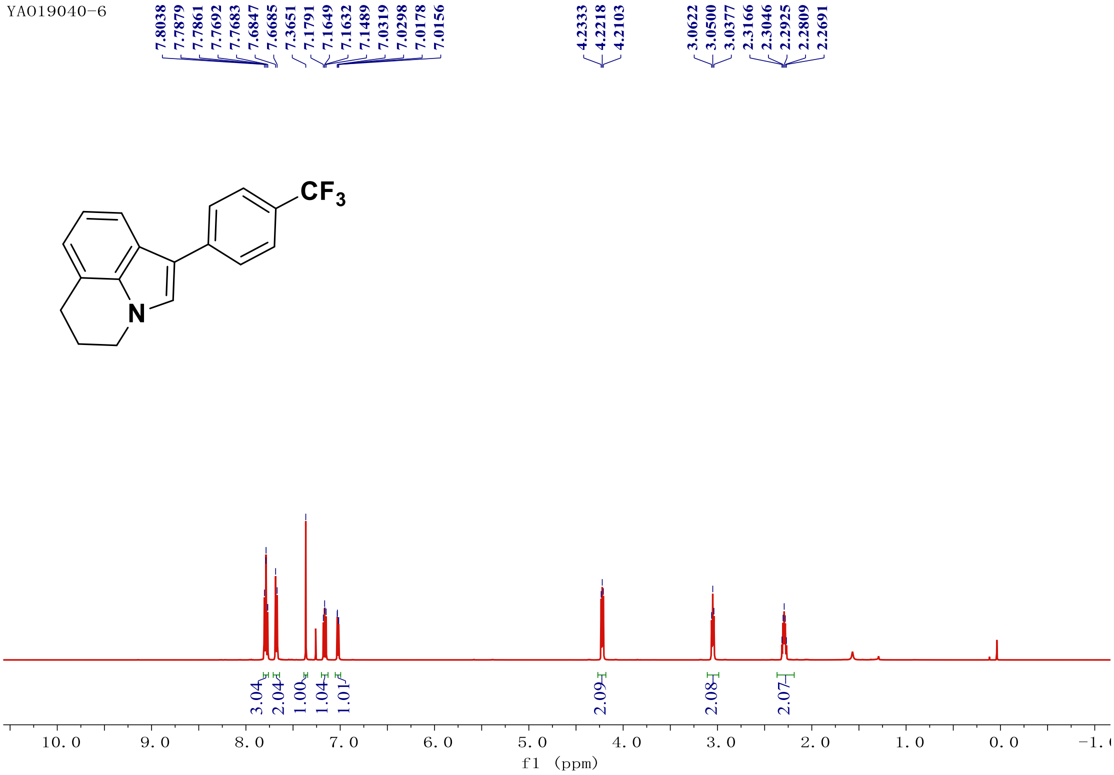

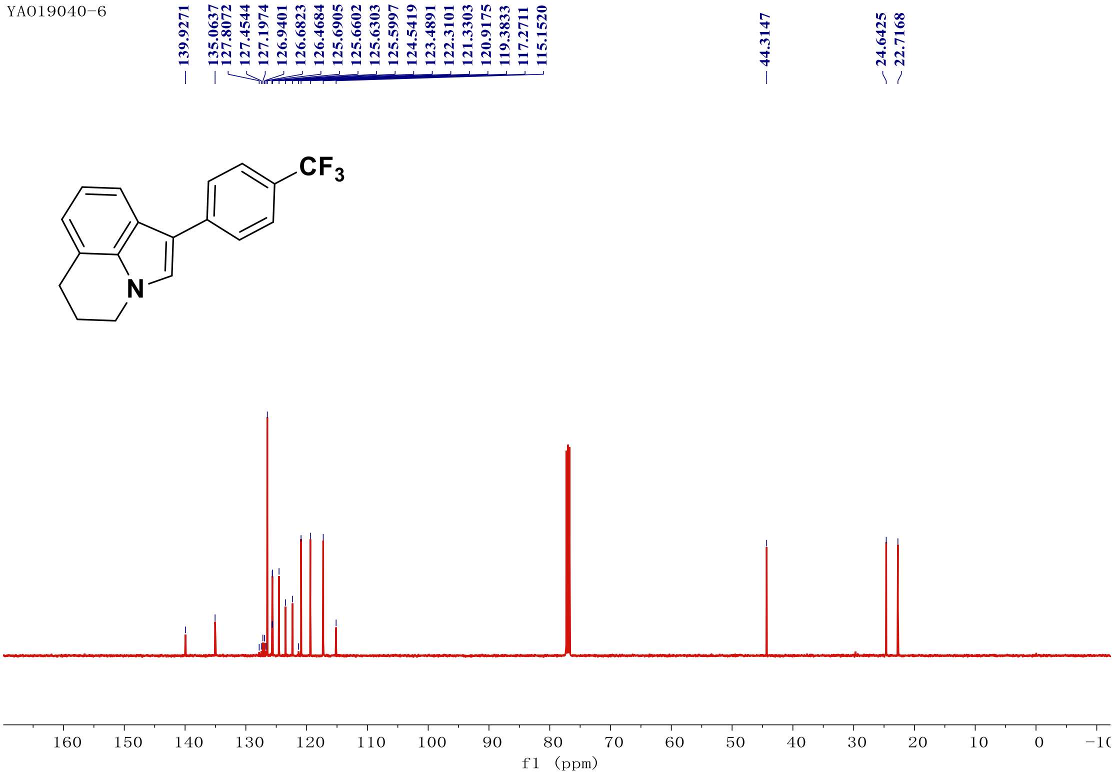

1-(4-Bromophenyl)-5,6-dihydro-4*H*-pyrrolo[3,2,1-*ij*]quinoline (**27**)

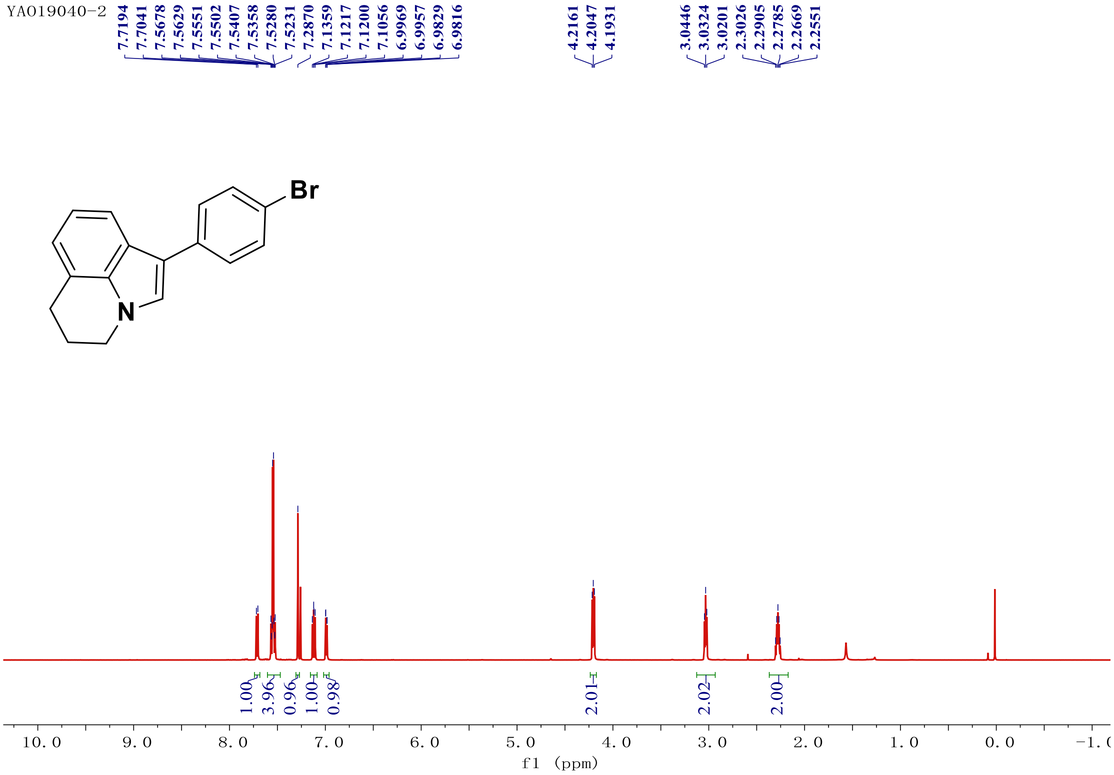

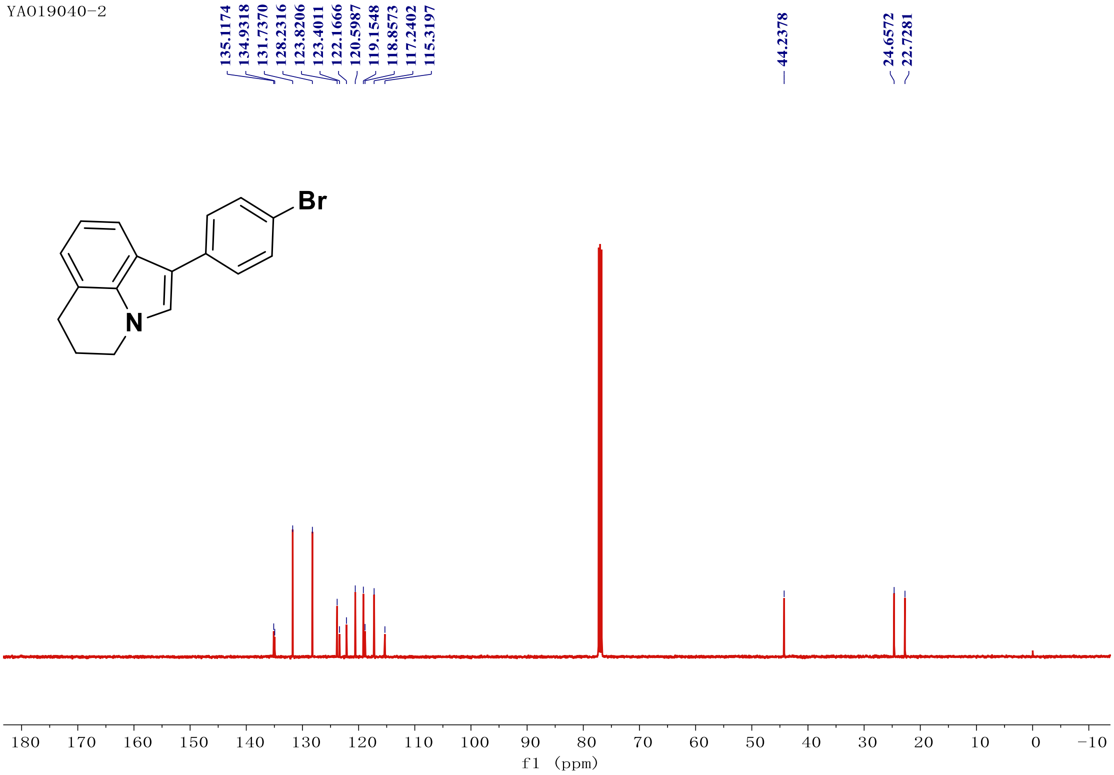

1-(4-Chlorophenyl)-5,6-dihydro-4*H*-pyrrolo[3,2,1-*ij*]quinoline (**28**)

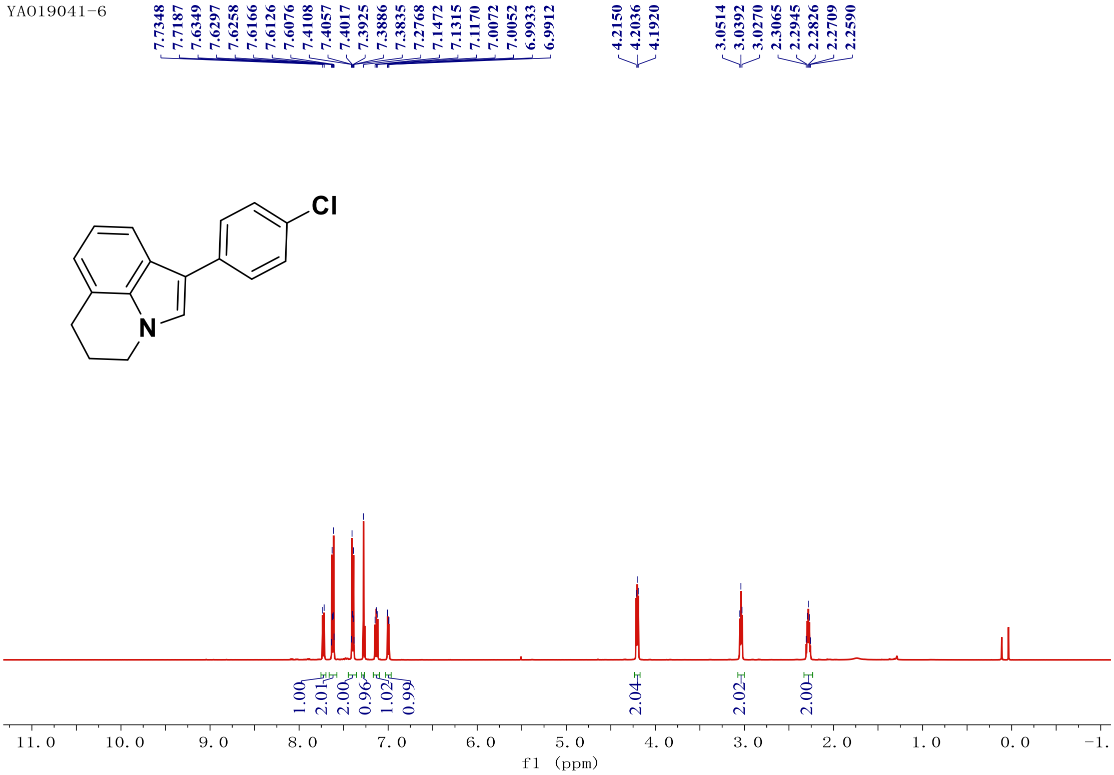

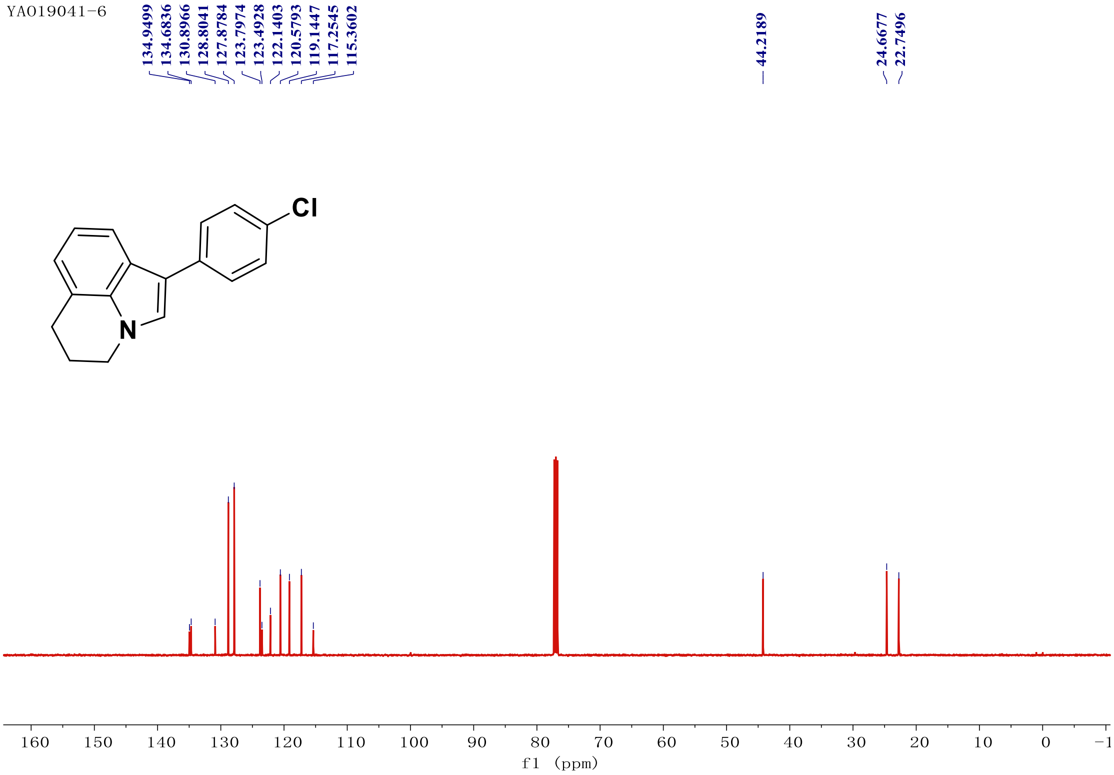

1-(4-Fluorophenyl)-5,6-dihydro-4*H*-pyrrolo[3,2,1-*ij*]quinoline (**29**)

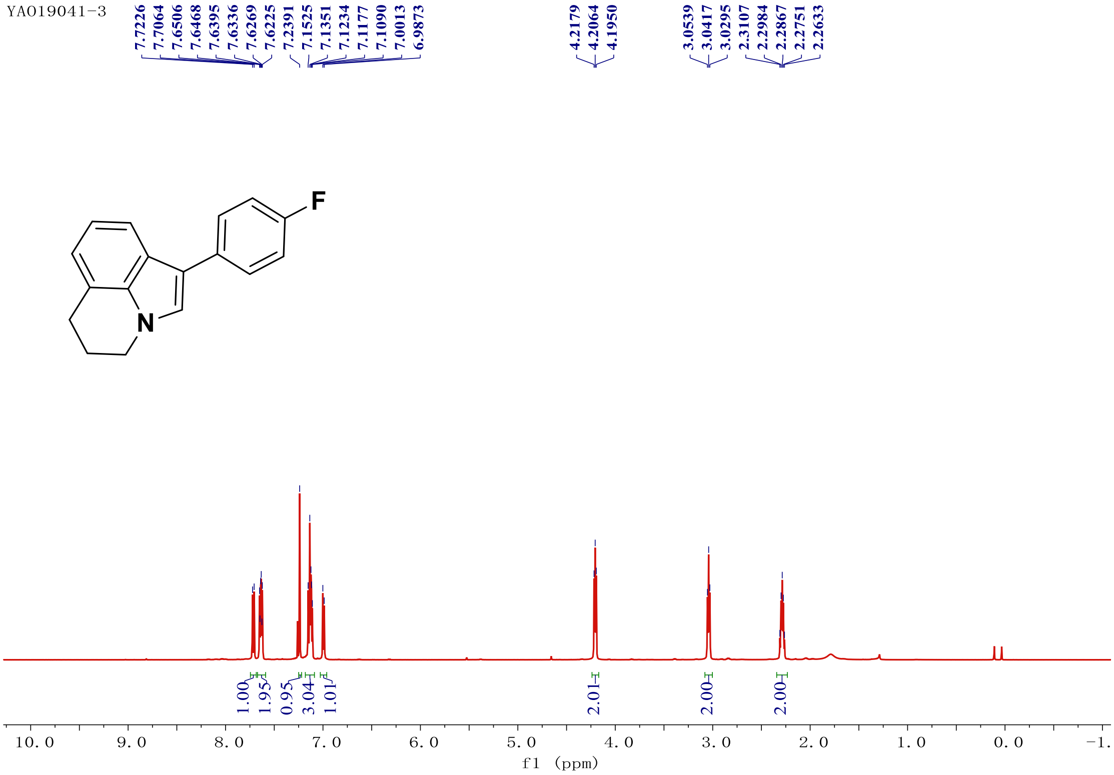

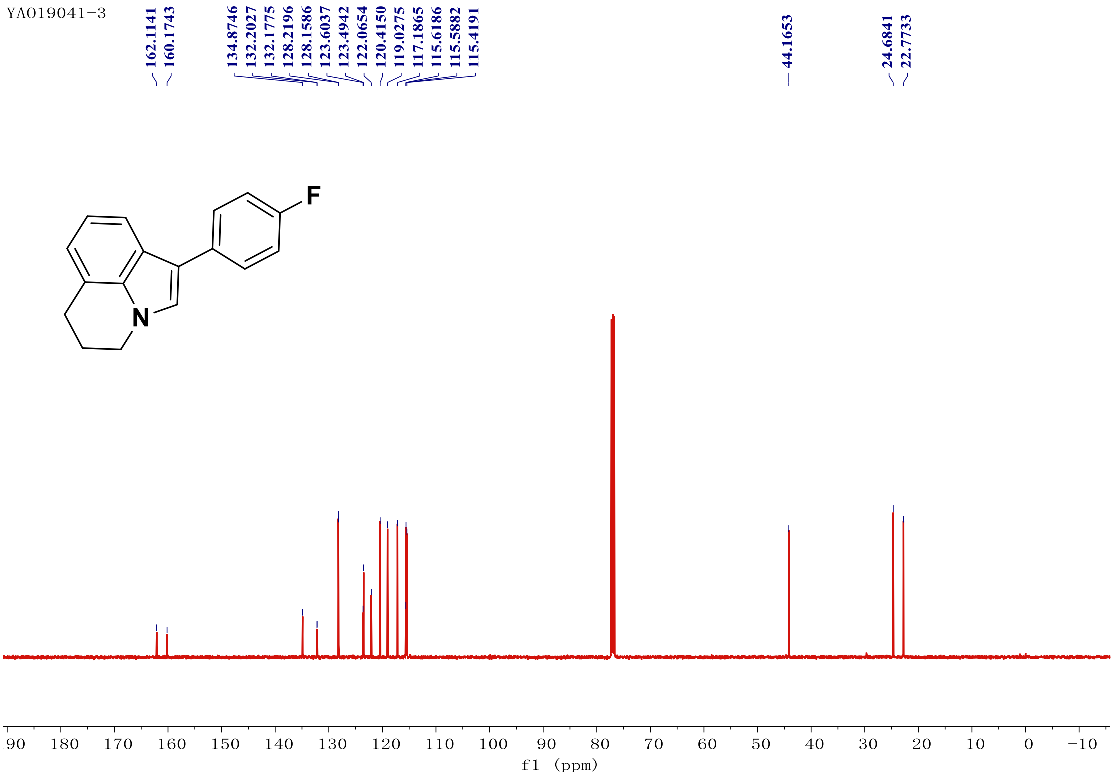

3-(5,6-Dihydro-4*H*-pyrrolo[3,2,1-*ij*]quinolin-1-yl)benzonitrile (**30**)

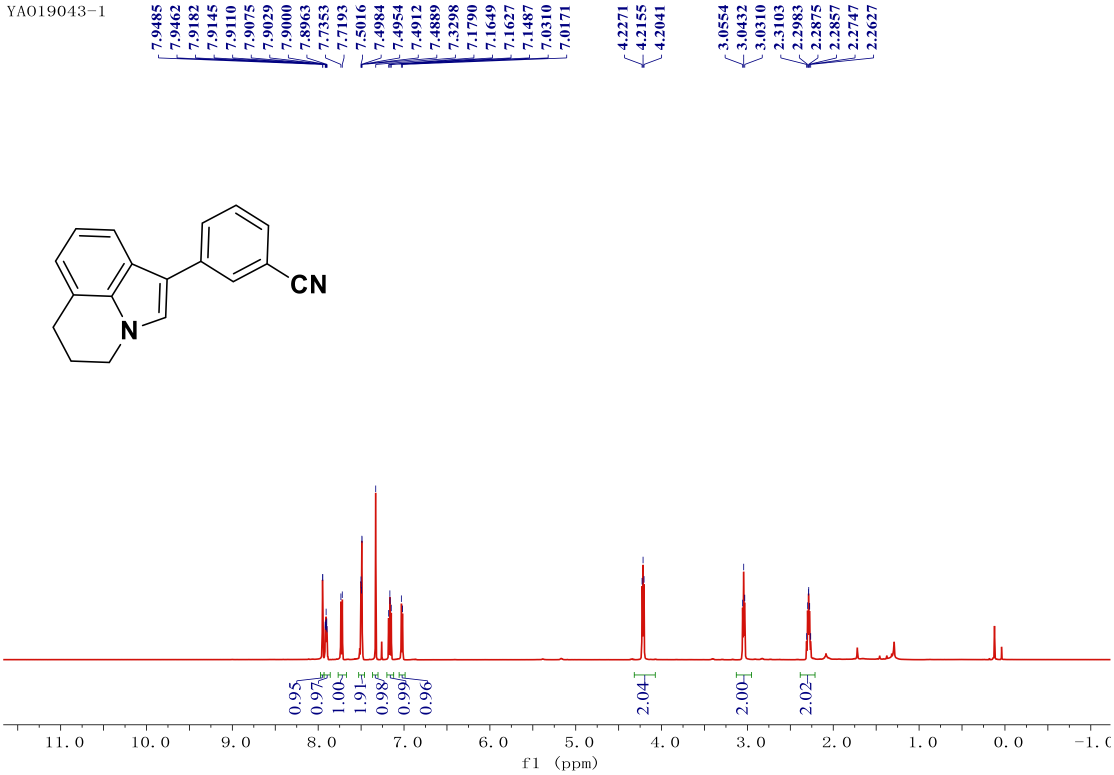

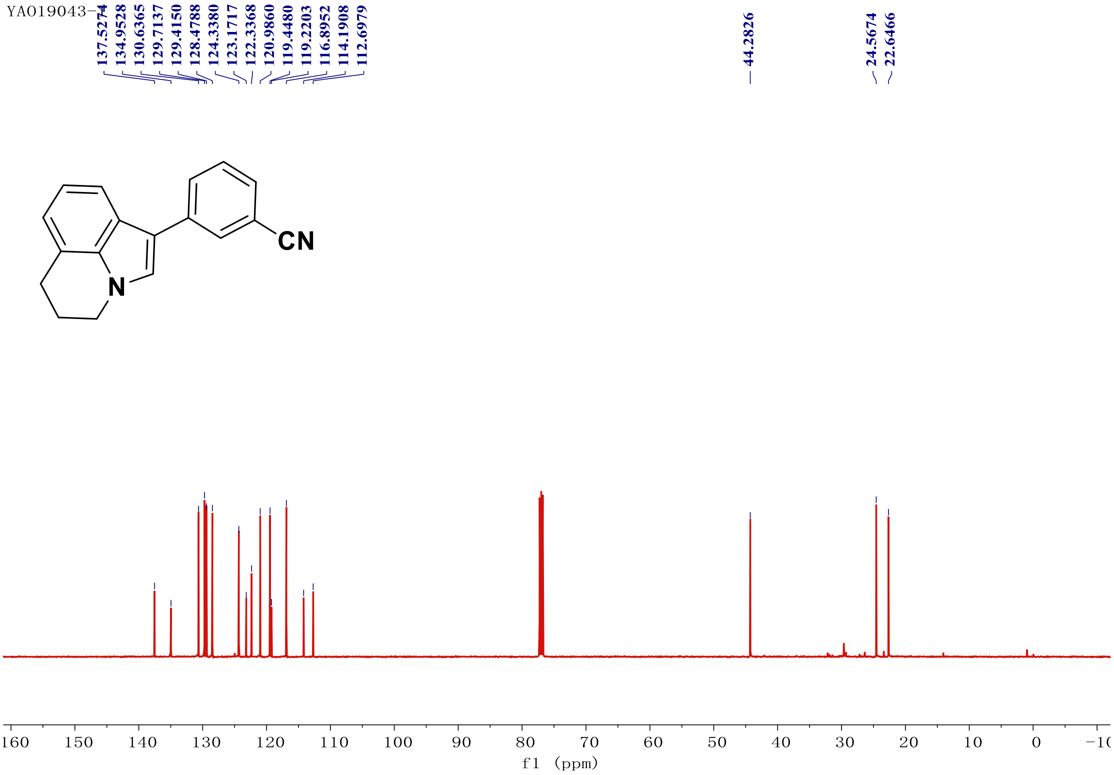

1-(Naphthalen-2-yl)-5,6-dihydro-4*H*-pyrrolo[3,2,1-*ij*]quinoline (**31**)

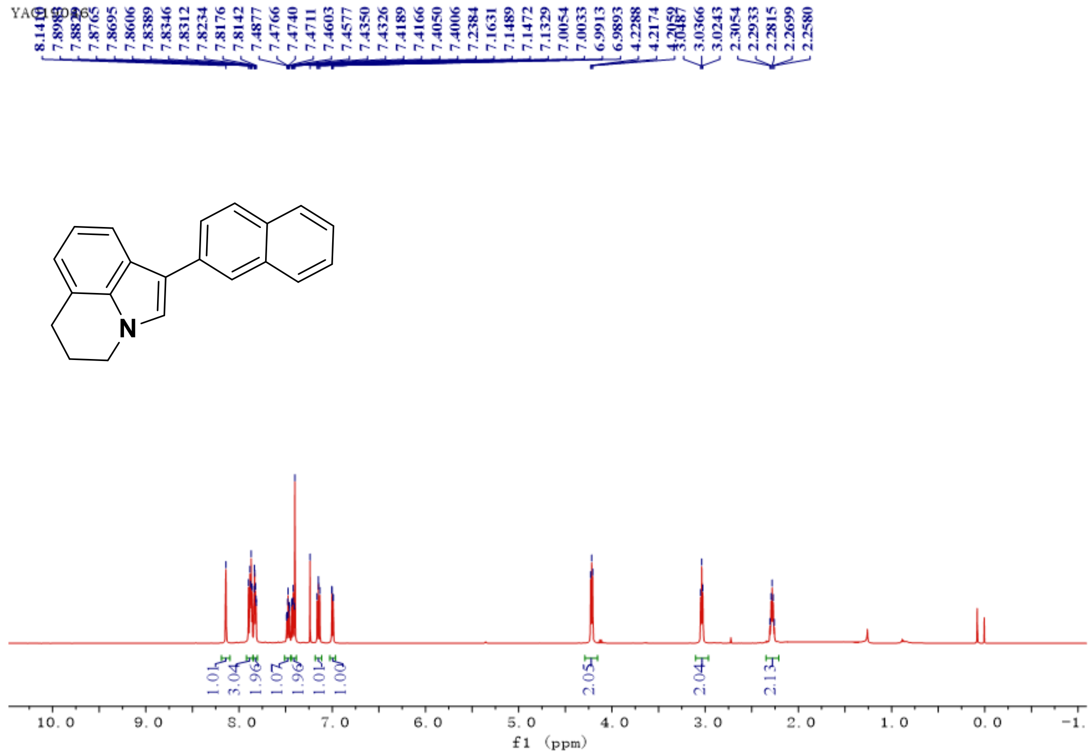

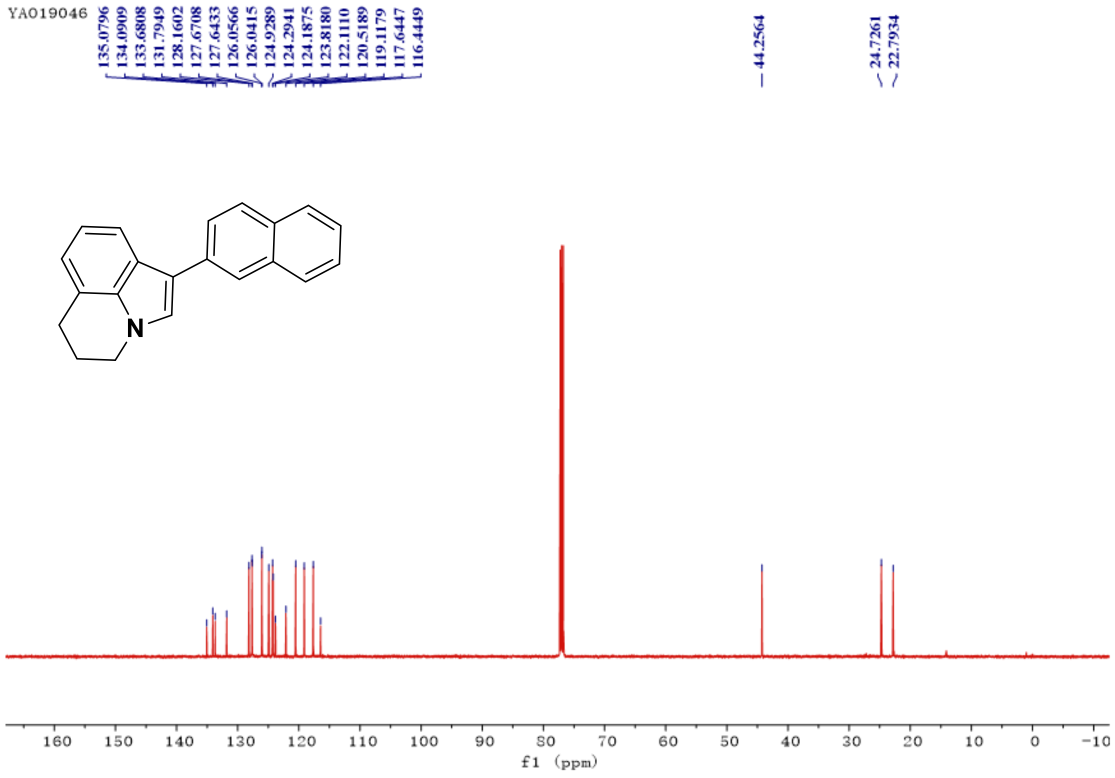

1-(Benzofuran-2-yl)-5,6-dihydro-4*H*-pyrrolo[3,2,1-*ij*]quinoline (**32**)

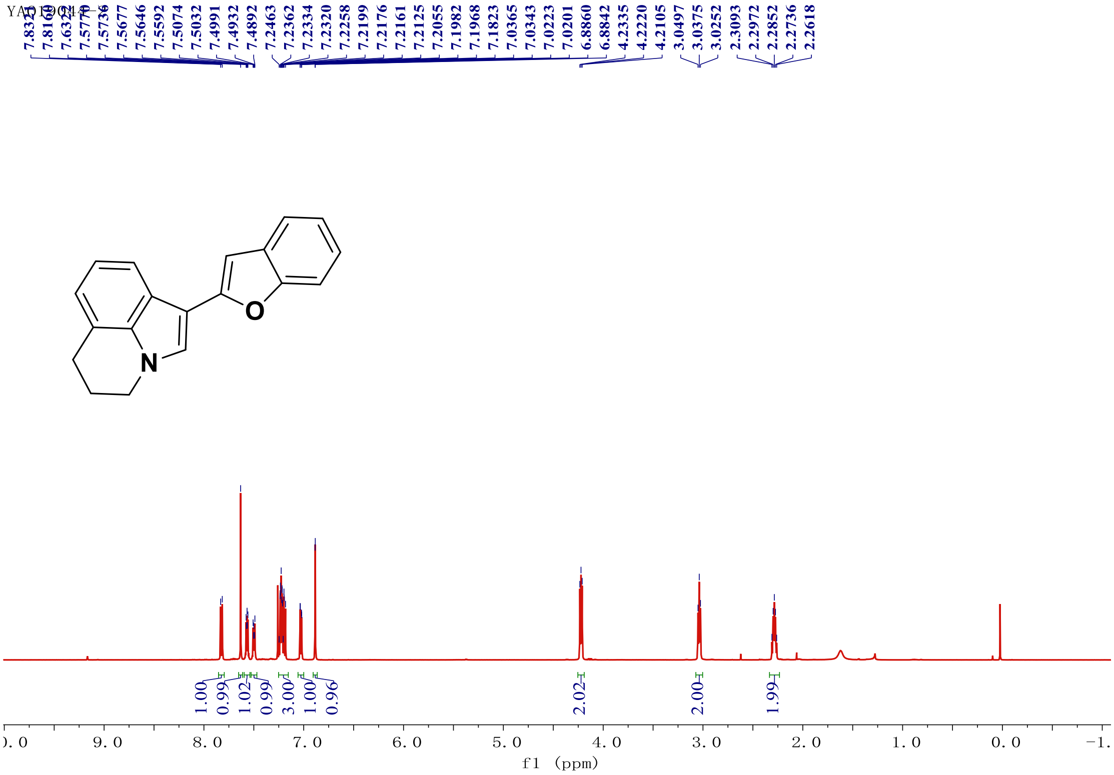

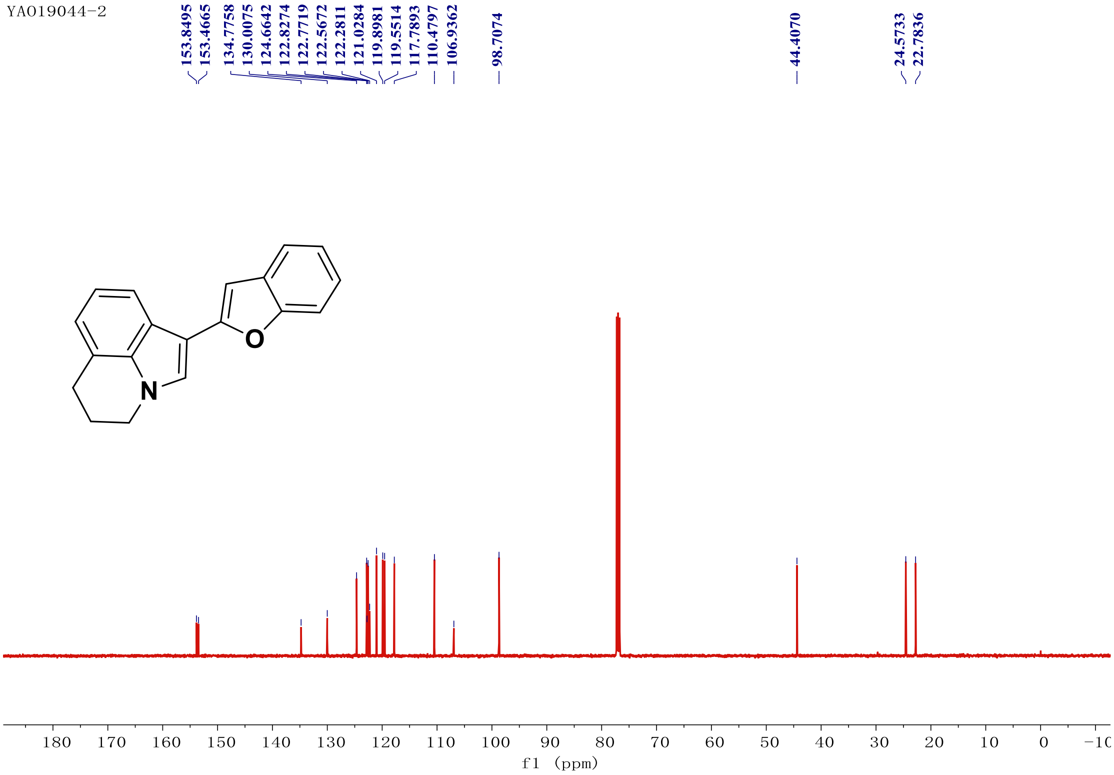

Ethyl 5,6-dihydro-4*H*-pyrrolo[3,2,1-*ij*]quinoline-1-carboxylate (**33**)

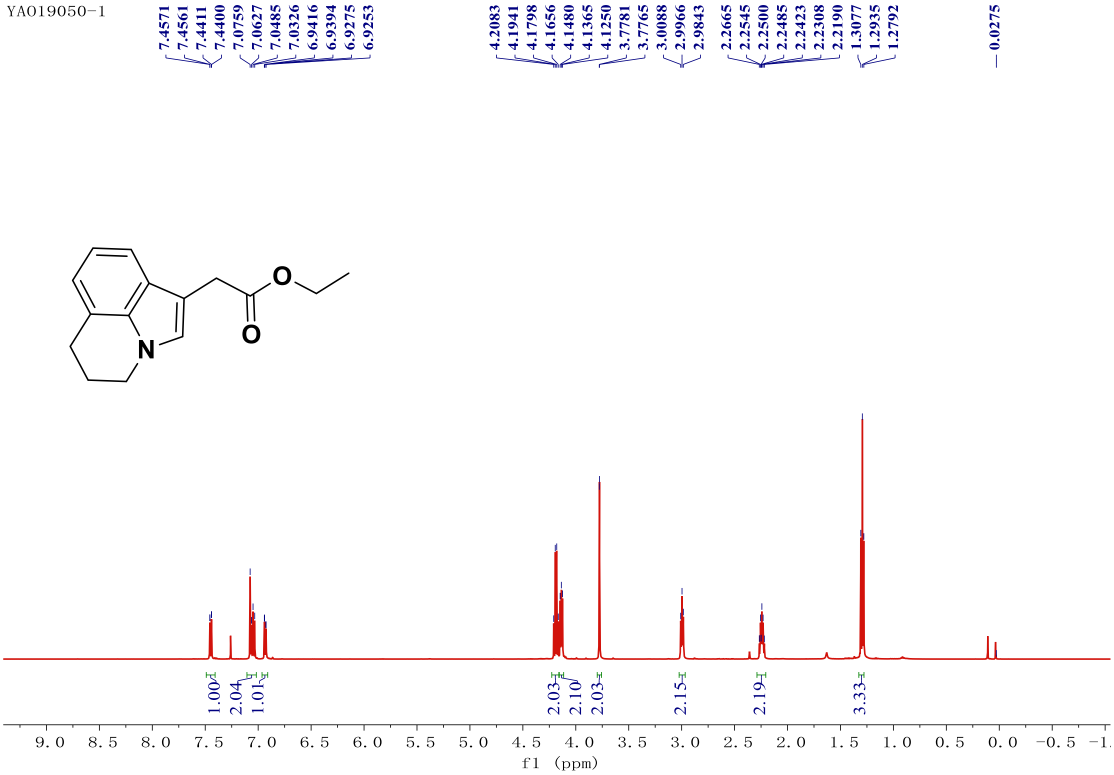

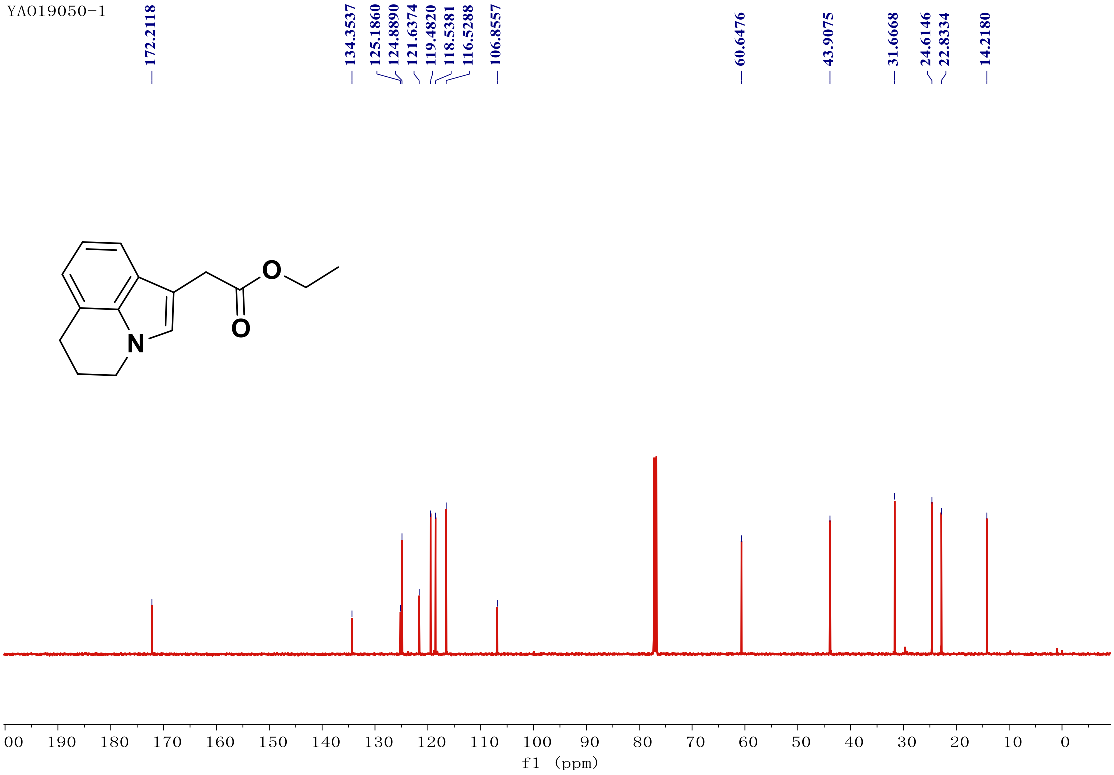

1-Ethyl-5,6-dihydro-4*H*-pyrrolo[3,2,1-*ij*]quinoline (**34**)

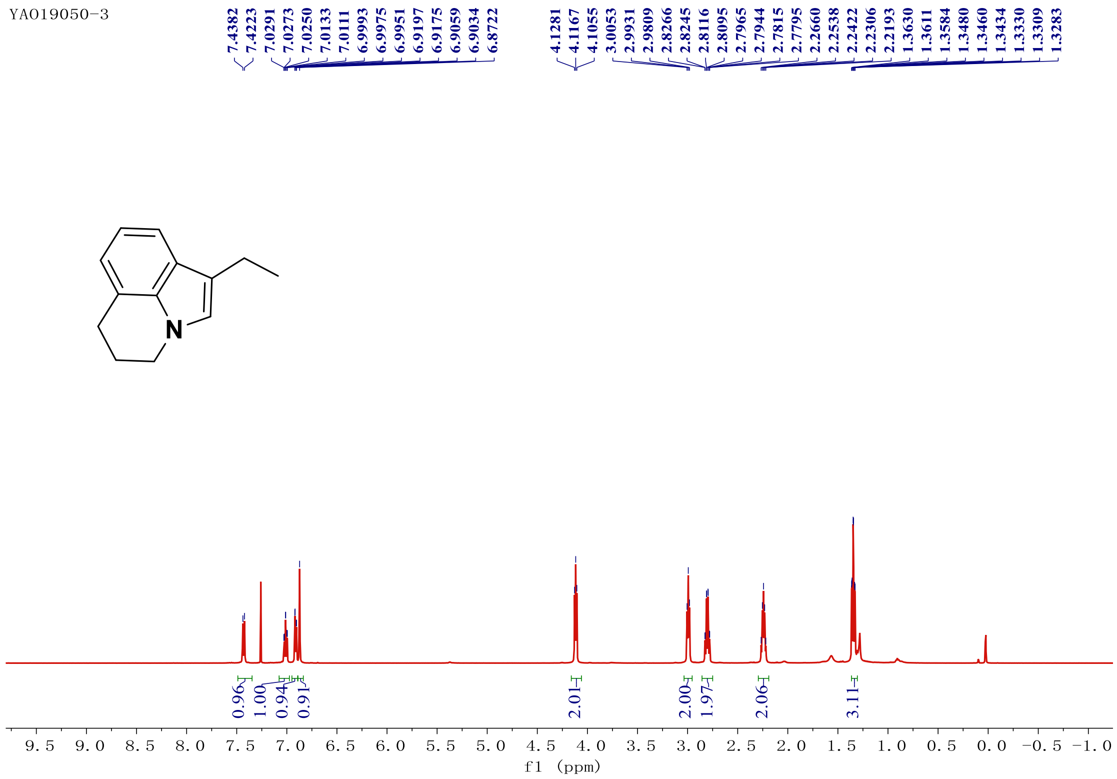

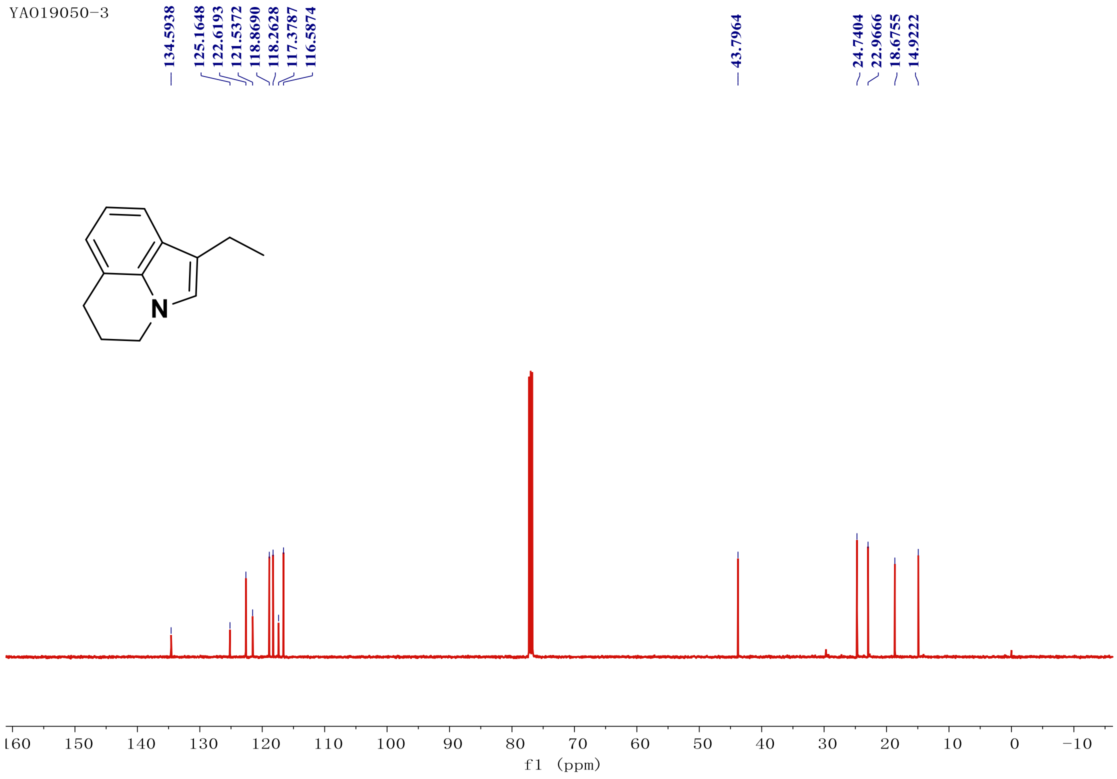

Supplement: Supplementary file 1 [file molecules-23-03317-s001.pdf]
